# Importance of balanced intracellular formate levels for pH_i_ and CO_2_ homeostases during mixed-acid fermentation

**DOI:** 10.1128/jb.00356-26

**Published:** 2026-09-03

**Authors:** Christopher Erdmann, Liana Vanyan, Karen Trchounian, R. Gary Sawers

**Affiliations:** 1 Institute for Microbiology, Martin Luther University Halle-Wittenberg 9176, Halle (Saale), Germany; 2 Department of Biochemistry, Microbiology and Biotechnology, Faculty of Biology, Yerevan State University 105430, Yerevan, Armenia; University of Florida, Gainesville, Florida, USA

**Keywords:** bicarbonate, CO_2_, fermentation, formate, formate hydrogenlyase, FocA

## Abstract

During mixed-acid fermentation, *Escherichia coli* metabolizes glucose to a mixture of acetate, ethanol, formate, lactate, and succinate, together with equimolar amounts of H_2_ and CO_2_. These gases are generated via the disproportionation of formate by formate hydrogenlyase (FHL-1). This reaction provides the cell with its only source of internally generated CO_2_ for carboxylation reactions. Formate is produced by pyruvate formate-lyase (PflB), and its balanced level within the cell is maintained by the activities of PflB, FHL-1, and the bidirectional formate channel, FocA. A formate imbalance results when strains synthesize a FocA_N209_ variant that continuously and efficiently exports formic acid. During fermentative growth, a FocA_N209_-producing strain prematurely enters stationary phase and fails to synthesize FHL-1 due to intracellular formate insufficiency. Here, we show that intracellular pH (pH_i_) of the mutant was increased relative to the parental strain, correlating with reduced ATP levels. Anaerobic growth could be partially restored by cultivation with bicarbonate; however, pH_i_ was lowered only marginally and ATP levels remained low. Determination of the fermentation-product profile revealed that the continuous loss of formate from the cell could not be compensated by bicarbonate supplementation. A severe limitation in both lactate and succinate production was also only partially rescued by bicarbonate. Increased ethanol production indicated that acetyl-CoA was sacrificed in the mutant to re-oxidize reduced pyridine nucleotides. These findings demonstrate that an imbalance in intracellular formate not only negatively impacts carboxylative metabolism but also remodels the spectrum of fermentation products to the detriment of the cell’s energy metabolism and pH homeostasis.

**IMPORTANCE:**

Formate is central to the fermentative metabolism of *Escherichia coli*. Balanced formate levels are required for DNA synthesis and for CO_2_ generation by formate hydrogenlyase (FHL-1). An *E. coli* mutant synthesizing an FocA variant that irreversibly exports formate has a severe growth defect because it cannot make FHL-1, which severely restricts CO_2_ and negatively impacts pH homeostasis by lowering ATP levels. We show, however, that impaired growth of the mutant can be only partially restored by supplying bicarbonate. The underlying cellular lack of formate cannot be fully compensated by bicarbonate because efficient pyruvate formate-lyase-dependent pyruvate cleavage remodels fermentation away from acetate and toward ethanol production, which ensures redox balance, but generates less ATP by substrate-level phosphorylation.

## INTRODUCTION

Mixed-acid-fermenting enterobacteria, such as *Escherichia coli,* are characterized by producing the enzyme pyruvate formate-lyase (PflB), which catalyzes cleavage of pyruvate into acetyl-CoA and formate (1, 2). The use of a non-oxidative, radical-based mechanism for pyruvate cleavage means that no CO_2_ results from this reaction, but equally, no reducing equivalents in the form of NADH are generated. Instead, the reducing equivalents, typically released during oxidative decarboxylation reactions, remain associated with the reaction product, formate. Formate is, however, subsequently disproportionated by the formate-inducible formate hydrogenlyase (FHL-1) complex, generating CO_2_ and releasing the reducing equivalents as H_2_. Thus, the FHL-1 reaction provides the cell with the CO_2_ it needs, which can be “captured” by carbonic anhydrase-catalyzed hydration to carbonic acid with subsequent intracellular deprotonation to anionic bicarbonate (3). The generation of the bicarbonate anion is favored at pH values above 7 and ensures that CO_2_ is not lost from the cell by diffusion (3). Bicarbonate, in turn, is a substrate for key carboxylation reactions, particularly those catalyzed by acetyl-CoA carboxylase during lipid biosynthesis (4), and by phosphoenolpyruvate (PEP) carboxylase, which generates oxaloacetate and allows re-oxidation of accumulated reducing equivalents by generating succinate as a fermentation product (2).

Formate generation is also essential for the anaerobic synthesis of deoxyribonucleotides, catalyzed by the glycyl-radical enzyme, anaerobic ribonucleotide reductase (5). As a consequence of the various functions of formate in metabolism, levels of the anion within fermenting *E. coli* cells must be carefully controlled. Balanced formate levels are achieved by the combined activities of formate-generating PflB, formate-consuming FHL-1, along with the bidirectionally functional formate channel protein, FocA. FocA translocates excess formic acid out of the cell during growth, but reimports it, as required, upon entry into the stationary phase. Re-import of formate is particularly important when the pH of the external environment decreases below 6.5, whereby FocA is proposed to deliver formic acid directly to FHL-1 for disproportionation (6, 7). As well as supplying the cell with CO_2_, this activity raises the external pH (pH_o_) because formate is translocated into the cell together with a proton, while this formic acid is then converted to neutral gaseous products, which readily diffuse across the cytoplasmic membrane and out of the cell. The overall consequence of these combined processes is the elevation of environmental pH and the maintenance of intracellular pH (pH_i_) and CO_2_ levels (8, 9).

A balanced mM concentration of intracellular formate (the p*K*_a_ of formic acid is 3.75) has the further consequence that the intracellular pH_i_ is around 6.8 (9, 10), which is considerably lower than the pH_i_ of 7.5 measured in aerobically growing cells (11), or, indeed, in the cytoplasm of some fermenting bacteria (12). The high formate level is, however, necessary for sensing by the formate-responsive transcriptional activator, FhlA, which once activated by formate induces the expression of the structural genes encoding the components of FHL-1 (13, 14).

As well as ensuring the removal of excess reducing equivalents from the fermenting cell as H_2_, and acting as a source of CO_2_, it was considered that FHL-1 might also function in conserving energy by translocating protons across the cytoplasmic membrane during the disproportionation reaction (6). This hypothesis was formulated because FHL-1 shares a common ancestry with the proton-translocating NADH dehydrogenase of the respiratory chain (6, 15, 16). Studies by the group of Frank Sargent have, however, shown that FHL-1 is unlikely to be able to translocate protons, mainly because the complex lacks three of the membrane-associated subunits that are important for proton translocation catalyzed by NADH dehydrogenase (6, 17). Nevertheless, through its role in helping maintain pH homeostasis, FHL-1 does contribute indirectly to energy conservation by maximizing ATP levels in fermenting cells. It does this by preventing excessive ATP hydrolysis catalyzed by the F_1_F_o_-ATPase, which concomitantly translocates protons into the periplasm to maintain the proton motive force (pmf) (9). Perhaps equally important with regard to the function of FHL-1 was the observation that generation of CO_2_ is also crucial in maintenance of pH_i_ and ATP levels (9). This was concluded based on an analysis of mutants lacking an FHL-1 complex, as they attain a lower final cell density and have a problem with stationary phase survival. Notably, these phenotypes of a *fhlA* mutant could be complemented by cultivation in the presence of sodium bicarbonate, which confirmed that the phenotypic deficiencies of FHL-1-negative strains were due to an inability to generate CO_2_ (9).

The phenotypes observed for mutants lacking FHL-1 are reminiscent of the strong fermentative growth phenotype exhibited by an *E. coli* strain that synthesizes a particular amino acid variant (FocA_N209_) of the formate-translocating FocA protein (18, 19). Pentameric FocA has two highly conserved amino acid residues within the pore of each of its five protomers (20, 21). A histidine residue at position 209 and a threonine residue at position 91 are both essential for controlled bidirectional translocation of formic acid through FocA (18, 22). While an exchange of T91 of FocA for an alanine residue causes a phenotype where the strain exhibits an impaired formic acid efflux during fermentation (22), an exchange of H209 for an asparagine causes the strain to export formic acid very efficiently (18). As well as a severe anaerobic growth deficiency shown to be characteristic of the mutant synthesizing FocA_N209_, the strain also produces virtually no H_2_ because the cytoplasmic formate concentration is maintained at a level that is insufficient for induction of FHL-1 synthesis (18). Here, we demonstrate that one reason for this negative growth phenotype is, indeed, due to restricted intracellular CO_2_ production similar to what is observed for an FHL-1-negative mutant (9). However, the severity of the growth and stationary phase survival phenotypes proved to be due to a remodeling of mixed-acid fermentation toward ethanol generation, caused by the continuous efflux of formic acid.

## RESULTS

### High bicarbonate concentration partially restores the fermentative growth defect of an *E. coli* strain synthesizing FocA_N209_

To ease comprehension, the strain names have been replaced by their phenotypic designations throughout the text, such that DH4100 is referred to as WT, DH4200 as FocAH209N, and DH4300 as FocAT91A.

**Fig 1:**
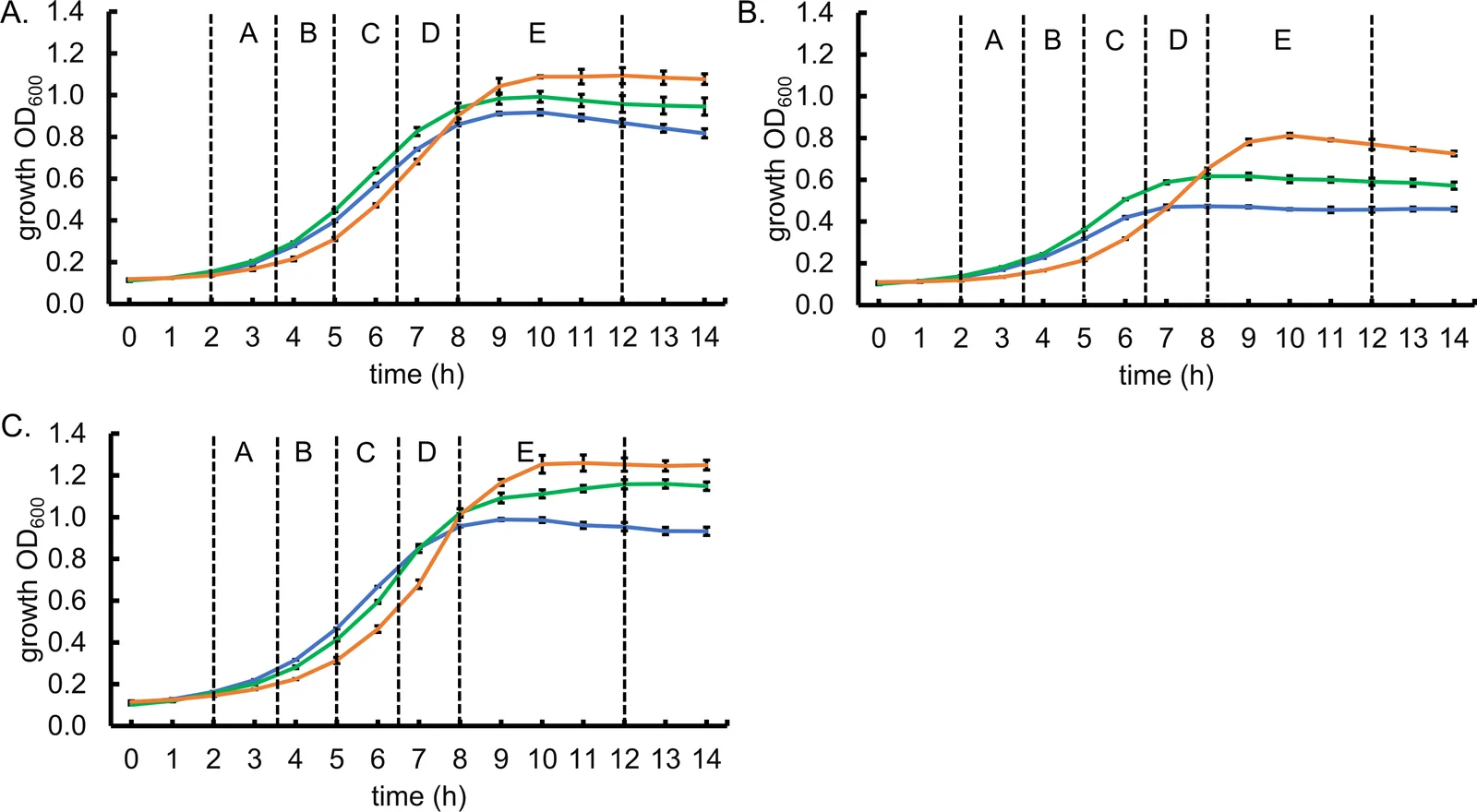
Growth during glucose fermentation of a mutant continuously exporting formic acid is severely restricted. Growth is represented with a linear *y*-axis to highlight the difference in final optical densities attained by the strains. The strains analyzed include DH4100 (WT, **A**); DH4200 (FocAH209N, **B**); DH4300 (FocAT91A, **C**). Each strain was grown anaerobically in M9-glucose medium, pH 7 (blue line), in M9-glucose medium, pH 8 (green line), or in M9-glucose medium, pH 7 supplemented with 50 mM sodium bicarbonate (orange line). The vertical dotted lines within each panel distinguish the lag-phase (A), the early (B), mid (C), and late (D) exponential growth phases and the stationary phase (E), and these are referred to when describing the rest of the data in the text. The growth experiments were performed with three biological and three technical replicates for each strain. Standard error of the mean is shown for each data point.

Strain FocAH209N (DH4200) has a chromosomal mutation in its *focA* gene at codon 209, which results in an exchange of the highly conserved histidine for an asparagine residue in the resultant FocA_N209_ protein (18). Despite being synthesized at wild-type levels in the cell, FocA_N209_ causes highly efficient translocation of formate from the cytoplasm into the periplasm (18). When anaerobic growth of FocAH209N in M9-glucose minimal medium at pH 7 was compared with the WT (DH4100) (μ = 0.31 ± 0.005 h^−1^; *n* = 3) (Fig. 1A), FocAH209N had a similar growth rate (μ = 0.3 ± 0.005 h^−1^) but showed a more rapid entry into stationary phase. However, FocAH209N only attained approximately half the final cell density of its parent (Fig. 1B; see Fig. S1 for corresponding semi-logarithmic plots) (18, 19).

The recent demonstration that *E. coli* mutants lacking a functional FHL-1 complex have a growth defect in late-exponential phase and show poor stationary-phase survival due to their inability to generate intracellular CO_2_ from formic acid disproportionation (9), suggested that insufficient levels of metabolically generated CO_2_ might at least partially account for the anaerobic growth defect observed for strain FocAH209N. Measurement of CO_2_ levels by gas-chromatography in the headspace of cultures of the WT and FocAH209N after 12 h of cultivation revealed that while 5.01 μmol of CO_2_ was measured in 200 mL of gas-phase of WT culture, only 0.32 μmol of CO_2_ was measured in the equivalent volume of headspace of FocAH209N cultures. This confirms that considerably lower levels of CO_2_ were produced by cultures of FocAH209N.

Supplementation of M9-glucose minimal medium, pH 7, with 50 mM sodium bicarbonate increased the specific anaerobic growth rate (μ = 0.36 ± 0.01 h^−1^) of FocAH209N and significantly increased the final OD_600_ (OD_600_ increase of 75%) attained by the culture (Fig. 1B). Analysis of the headspace of the culture revealed the presence of 9.2 μmol of CO_2_ in 200 mL of gas volume. It was noted, however, that high bicarbonate concentration extended the lag-phase of FocAH209N by approximately 2 h, compared to cultivation without bicarbonate (Fig. 1B; Fig. S1). As a control, supplementation of cultures of the WT with 50 mM bicarbonate similarly caused a lengthening of the lag-phase, while the anaerobic growth rate (μ = 0.36 ± 0.01 h^−1^) remained similar to that of FocAH209N. An increase in the final cell density of 0.2 units was also determined after growth with bicarbonate, relative to growth without bicarbonate, for the WT culture (Fig. 1A). The amount of CO_2_ in the headspace was 5.4 μmol per 200 mL of headspace volume.

Despite the fact that increased bicarbonate in the culture medium of FocAH209N improved growth, the final OD_600_ attained by the formate-export mutant was still lower than that of the WT grown under the same conditions (compare Fig. 1A and B). This result indicated that growth of FocAH209N was restricted, presumably by the loss of formate from the cell.

Supplementation of 50 mM sodium bicarbonate to the growth medium also causes an approximate 0.5 unit increase in the pH of the M9 medium (9). Therefore, to exclude that the positive effect of bicarbonate supplementation on growth of FocAH209N was the result of an increase in pH of the culture medium, cultures of WT and FocAH209N were grown in M9-glucose medium at an initial pH of 8 and compared with each other (Fig. 1A and B). No extended lag-phase was observed for either culture under this condition, which contrasted what was observed for growth with 50 mM bicarbonate. While the growth rate of both strains at pH 8 was improved, by 6% for WT (μ = 0.33 ± 0.02 h^−1^) and by 20% for FocAH209N (μ = 0.36 ± 0.08 h^−1^); nevertheless, the increase in final optical density of the respective cultures was considerably lower than that attained after supplementation with bicarbonate (Fig. 1A and B). This indicates that the positive effect of adding sodium bicarbonate on fermentative growth of FocAH209N was not exclusively due to an increase in initial pH of the growth medium, but rather indicated that it was the result of an increased intracellular bicarbonate/CO_2_ concentration, as observed recently for a *fhlA* mutant (9). Notably, supplementation of the culture medium with either 10 mM (Fig. S2) or 50 mM bicarbonate resulted in a similar maximal increase in final cell density of cultures of the WT (Fig. S2). In contrast, cultures of FocAH209N supplemented with 10 mM bicarbonate showed only a limited improvement in growth (Fig. S2A) when compared with addition of 50 mM bicarbonate (Fig. 1B).

**Fig 2:**
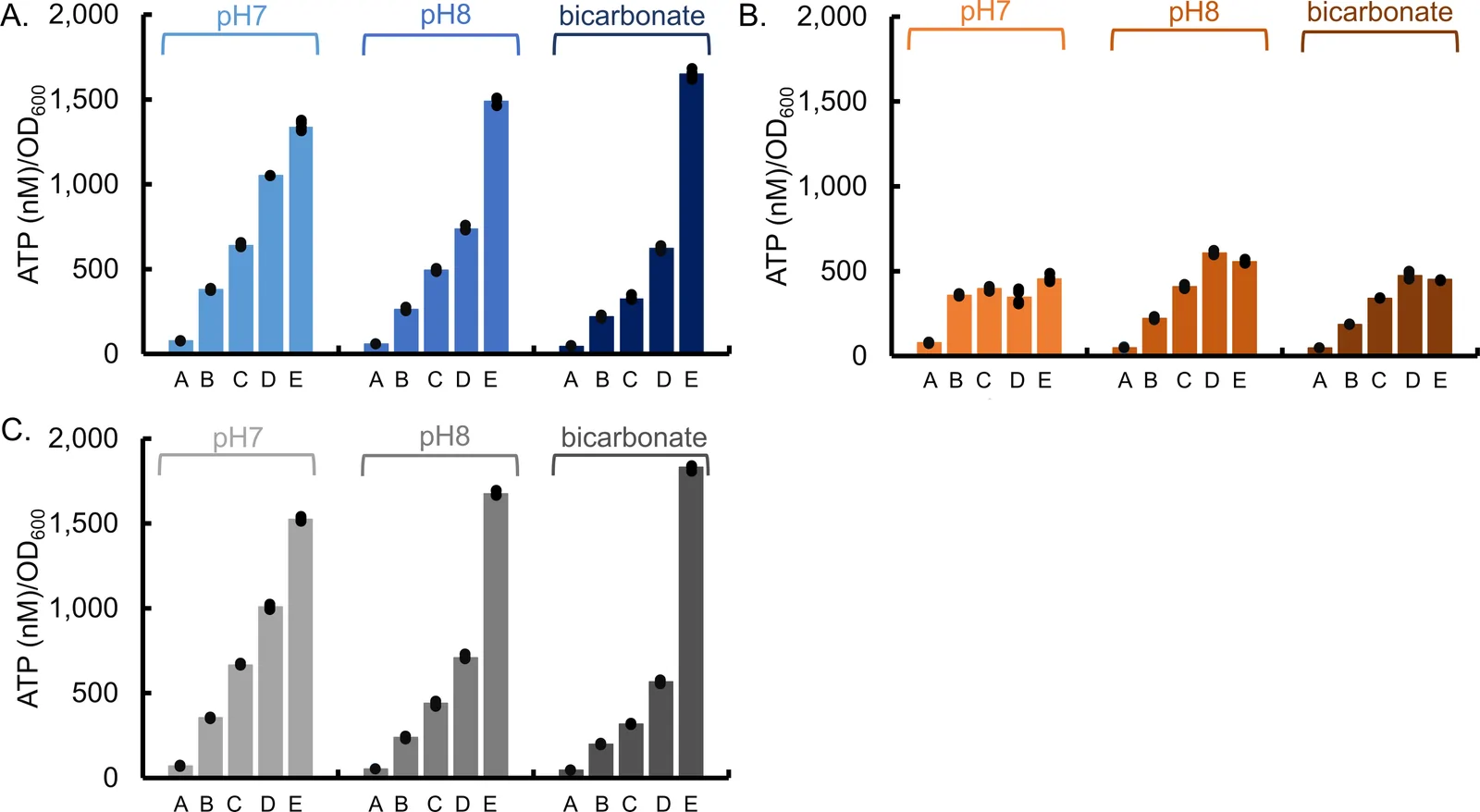
ATP concentrations determined throughout the growth phase during glucose fermentation. Each panel (**A**) (WT, DH4100), (**B**) (FocAH209N, DH4200), and (**C**) (FocAT91A, DH4300) shows a series of five histograms depicting the ATP levels in the growth phases A through E, which are indicated on the *x*-axes, and correspond to the growth phases shown in Fig. 1. The times at which samples were taken for determination of ATP were as follows: phase A, ~3 h; phase B, ~4 h; phase C, ~6 h; phase D, ~8 h; phase E, 12 h. Growth was done in M9-glucose minimal medium, at pH 7, pH 8, or pH 7 plus 50 mM bicarbonate as indicated above each set of histograms. Data points are shown for three biological replicates, with the standard error of the mean shown.

Together, these results demonstrate that although the addition of 50 mM bicarbonate to anaerobic cultures of FocAH209N significantly improved fermentative growth of the strain, this growth improvement did not match that observed for the WT strain. These data suggest that the high efflux of formate (18) is likely responsible for the growth limitation of FocAH209N.

### An *E. coli* strain with a FocA-dependent deficiency in export of formate grows like the parental strain

Together, the results of the growth experiments presented above suggest that the lack of CO_2_ and formate in strain FocAH209N, which efficiently exports formic acid, is the reason for the anaerobic growth restriction of the strain. If this contention is correct, then a strain with the opposite phenotype that accumulates formate intracellularly should show a weaker response to bicarbonate supplementation and should not show a reduced growth rate during fermentation. Strain FocAT91A (DH4300) synthesizes a FocA_A91_ variant that has an impaired ability to translocate formic acid across the cytoplasmic membrane and accumulates the anion in the cytoplasm (22). Strain FocAT91A grew in M9-glucose medium with an anaerobic growth rate (μ) of 0.33 ± 0.03 h^−1^ and a slightly higher final cell density compared with the WT grown under the same conditions (Fig. 1C). While the growth rate of strain FocAT91A was further increased at pH 8 (μ = 0.37 ± 0.01 h^−1^), it was increased still further after supplementation of the medium with 50 mM sodium bicarbonate (μ = 0.4 ± 0.01 h^−1^). Nevertheless, although growth of FocAT91A was slightly higher than that of WT, the effects of both growth at pH 8 and supplementation with bicarbonate were qualitatively similar for both strains. Moreover, as observed for the WT, anaerobic cultivation of FocAT91A in the presence of 10 mM bicarbonate led to a similar increase in final cell density to that observed for growth in the presence of 50 mM bicarbonate (Fig. S2). The statistical significance (*P* < 0.001) of the growth differences between the three strains under the different conditions analyzed is summarized at the end of the Supplementary Material (Statistics Table S1).

Together, these growth data strongly suggest that if intracellular formate levels are maintained at a similar, or even higher, level than the WT, then this likely results in maintenance of pH_i_ in the range of 6.8 (9; and see below) and consequently limits the impact of bicarbonate-supplementation on fermentative growth.

### ATP concentrations in strain FocAH209N (DH4200) are not restored to wild-type levels during anaerobic cultivation with 50 mM bicarbonate

ATP levels during fermentative growth in M9-glucose medium have recently been measured in cells of both the WT (9, 19) and FocAH209N during fermentation (19); however, a more detailed determination for both strains is shown in Fig. 2A and B. The results revealed that the amount of ATP determined in FocAH209N reached a plateau of 400 nM OD_600_^−1^ between 4 h and 6 h of growth. This correlates with an early transition into the stationary phase of FocAH209N at around 6 h (Fig. 2B; see also Fig. 1B, phase C). Such an early entry into stationary phase does not occur with the WT, whereby a steady increase in the ATP level continued into the stationary phase, with a final ATP level of 1.4 μM OD_600_^−1^ being determined (Fig. 2A; phase E, 12 h).

Anaerobic cultivation of WT in M9-glucose medium at an initial pH of 8 resulted in a minor increase in the amount of ATP (1.5 μM OD_600_^−1^) in phase E (stationary phase) compared with cultivation at pH 7 (Fig. 2A). However, although stationary-phase cells of strain FocAH209N had a slightly higher ATP level of 600 nM OD_600_^−1^ after growth at pH 8, compared with growth at pH 7 (Fig. 2B, phases D and E), this amount was considerably lower than that measured in the WT (Fig. 2A).

Anaerobic cultivation of WT in M9-glucose medium, pH 7, supplemented with 50 mM sodium bicarbonate resulted in an ATP concentration of 1.6 μM OD_600_^−1^ after 12 h (Fig. 2A, phase E), while the levels in phases B, C, and D were lower by ~10%, 40%, and 43%, respectively, compared with the level measured in cells grown without added bicarbonate (Fig. 2A). Despite anaerobic growth of strain FocAH209N being significantly improved by bicarbonate-supplementation (see Fig. 1B), nevertheless, ATP levels were essentially unaffected under these conditions and remained at a level similar to that measured during cultivation without bicarbonate supplementation (Fig. 2B).

The ATP levels determined in cells of FocAT91A cultivated in M9-glucose medium at pH 7, at pH 8, and after supplementation with 50 mM bicarbonate (Fig. 2C) were very similar throughout growth to those measured for the WT (compare Fig. 2A). The exception was that under all of these growth conditions, the concentration of ATP after 12 h of cultivation (phase E) was approximately 10% to 15% higher compared to the level determined for the WT (compare Fig. 2A and C and see Statistics Table S2). This higher ATP level possibly explains why strain FocAT91A attained a higher final cell density than its parent (compare Fig. 1A and C).

### FocAH209N (DH4200) has a constant pH_i_ of 7.0 during glucose fermentation

**Fig 3:**
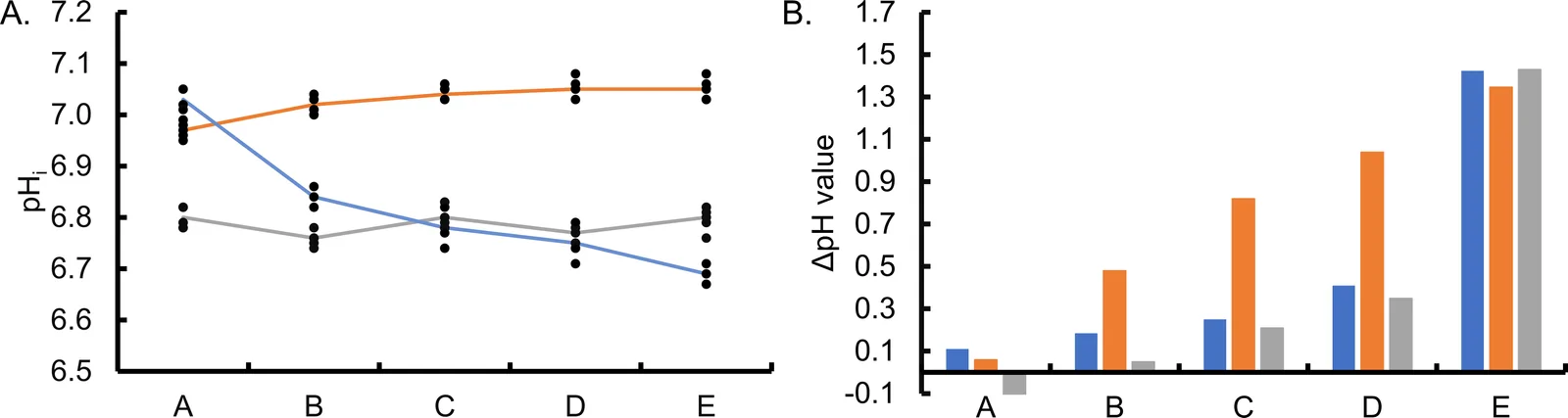
Comparison of internal and ΔpH values during glucose fermentation of strains synthesizing FocA variants. Panel **A** shows the cytoplasmic pH (pH_i_) determined for the WT (DH4100—blue line), FocAH209A (DH4200—orange line), and FocAT91A (DH4300—gray line) during the different growth phases, as indicated in the legend to Fig. 2, after cultivation in M9-glucose medium, pH 7. Panel **B** shows the ΔpH (pH_i_–pH_o_) determined for the same strains during the different phases of growth. The values of pH_o_ are shown in Fig. S3A. Determination of pH_i_ and pH_o_ was done for three biological replicates each.

During fermentative growth with glucose, the pH_i_ of the WT was determined to be approximately 7 at the beginning of growth, and then, it decreased to around 6.8 during the exponential growth-phase, before attaining a pH of 6.7 in stationary phase (9; Fig. 3A, blue curve). In contrast, during cultivation of strain FocAH209N (synthesizing FocA_N209_), which maintains a low intracellular formate concentration (18), the pH_i_ attained a level slightly above 7 and maintained this level constantly throughout all phases of growth, including into the stationary phase (Fig. 3A, orange curve).

Determination of the pH_i_ of strain FocAT91A, which has elevated intracellular formate levels during fermentative growth (22), revealed that a constant pH_i_ of 6.8 was maintained throughout all phases of growth (Fig. 3A, gray curve; Statistics Table S3). These data confirm recent findings showing that a pH_i_ of approximately 6.8 is characteristic for optimal exponential growth of WT *E. coli* by mixed-acid fermentation of glucose (9, 10). In contrast, strains lacking an active FHL-1 complex hydrolyze ATP to extrude protons via F_1_F_o_-ATPase, which raises the cytoplasmic pH to just above 7 (9). Thus, the inverse correlation between low ATP levels and a correspondingly higher pH_i_ for strain FocAH209N during glucose-fermentation is consistent with a broadly similar phenomenon occurring to that shown recently for the FHL-1-negative mutants.

### Increased activity of F_1_F_o_-ATPase in strain FocAH209N (DH4200)

The total ATPase activity and the activity due exclusively to the F_1_F_o_-ATP synthase (F_1_F_o_-ATPase) were determined in membrane vesicles derived from the WT and strains FocAH209N and FocAT91A. Measurements were made after anaerobic cultivation of the strains in M9-glucose medium to phase C (mid-exponential phase) and to phase E (stationary phase) (Table 1). The results show that the total ATPase activity of vesicles from exponential-phase cells (phase C) of FocAH209N was significantly higher by 2.5-fold than that in vesicles of FocAT91A (Table 1) and 2-fold higher than that of WT, while the F_1_F_o_-ATPase contribution was 3-fold and 2-fold higher than for vesicles from FocAT91A and WT, respectively. Membrane vesicles derived from stationary-phase cells (phase E) of FocAH209N had a total ATPase activity that was 2-fold higher than vesicles derived from FocAT91A (Table 1), and the contribution of the F_1_F_o_-ATPase in membrane vesicles from FocAH209N was 2.4-fold higher. The comparison with vesicles from WT (9) showed there was a nearly 3-fold higher total ATPase activity in vesicles from FocAH209N and a 3.7-fold higher F_1_F_o_-ATPase contribution. These activity data are consistent with the considerably lower ATP concentration in cells of FocAH209N during cultivation by fermentation.

**TABLE 1:** ATPase activity in membrane vesicles

Strain and growth phase^*a*^	Total ATPase activity (nmol Pi min-1 µg-1)	Total ATPase activity + DCCD (nmol Pi min-1 µg-1)	Activity due to F1Fo-ATPase (nmol Pi min-1 µg-1)
WT (phase C)^*b*^	176 ± 25	42 ± 15	134 ± 29
WT (phase E)^*b*^	135 ± 35	56 ± 5	79 ± 35
FocAH209N (phase C)	346 ± 10 ****	83 ± 8	263 ± 13****
FocAH209N (phase E)	388 ± 5 ****	94 ± 15	294 ± 16****
FocAT91A (phase C)	137 ± 23	55 ± 11	82 ± 26^*c*^
FocAT91A (phase E)	189 ± 15^*c*^	67 ± 13	122 ± 20

^*a*^ Growth phases C and E represent late-exponential phase (6 h) and stationary phase (12 h), respectively (see Fig. 1).

^*b*^ Data reported in reference 9.

^*c*^ Values are presented as mean ± SD (n = 3). Statistical analysis was performed using two-way ANOVA followed by Tukey's multiple comparisons test. Asterisks denote significant differences compared with the corresponding wild-type strain (DH4100) at the same growth phase (*P < 0.05, ****P < 0.0001); values without asterisks indicated that no significant difference was observed.

### Increased ΔpH in strain FocAH209N (DH4200) during growth by glucose fermentation is compensated by an adjusted ΔΨ

Determination of pH_o_ (Fig. S3) and pH_i_ (Fig. 3A) values for strains FocAH209N and FocAT91A during anaerobic cultivation revealed a 3- to 5-fold increase in ΔpH (pH_i_-pH_o_) in strain FocAH209N in the exponential growth phase (Fig. 3B; phases B through D) compared with FocAT91A and with WT. However, in stationary-phase cells, the ΔpH of approximately 1.3 pH units was similar for all three strains (Fig. 3B; phase E).

Determination of the membrane potential (ΔΨ; Table S1) in cells of all three strains (see Methods) after cultivation for 6 h (mid-exponential phase; phase C in Fig. 1) and 12 h (stationary phase; phase E) revealed that in phase C, the membrane potential for all three strains was in the range of −93 to −99 mV (Table S1). In stationary-phase cells (12 h), the ΔΨmeasured for both WT and FocAH209N was approximately −130 mV, while the ΔΨ measured for FocAT91A was 50 mV more negative (Table S1). The calculated proton motive force (pmf) across the cytoplasmic membrane of exponentially growing cells revealed that, due to the higher ΔpH in FocAH209N, the pmf was 33 mV more negative compared with the level in the WT and was 42 mV more negative than in FocAT91A. In stationary phase cells, the pmf was −221 and −213 mV for WT and FocAH209N, respectively, but was higher, at −265 mV, for strain FocAT91A (Table S1). Together, these data indicate that the differences measured in the ΔpH and ΔΨ between the strains in the two cultivation phases were largely compensated by each other, such that the pmf was maintained at minimally −200 mV, especially in stationary-phase cells of all three strains.

### Effect of bicarbonate supplementation on pH_i_ in the formate-efflux mutant, FocAH209N (DH4200)

After cultivation of FocAH209N for 12 h (phase E in Fig. 1B) in the presence of 50 mM sodium bicarbonate, the pH_i_ was determined to be 6.92 (Table S2), which was midway between that determined for the same strain (pH_i_ = 7.0) cultivated without bicarbonate supplementation and that determined for FocAT91A (pH_i_ = 6.8), also when cultivated at pH 7 (compare Fig. 3A). The presence of 50 mM bicarbonate was thus unable to restore fully either parental growth, or ATP levels to FocAH209N (see Fig. 1 and 2). This suggests that an increase in pH_i_ of 0.1 unit (above pH_i_ = 6.8) was sufficient to induce ATP hydrolysis in the strain (Table 1), and which was shown previously to be the main cause of reduced ATP levels in FHL-1-negative mutants (9).

Determination of the pH_i_ in WT and FocAT91A after 12 h of anaerobic cultivation in M9-glucose medium supplemented with 50 mM bicarbonate yielded levels of 6.8 and 6.82, respectively (Table S2). These pH_i_ levels correlate with the respective higher intracellular ATP levels in these strains (see Fig. 2A and C) and appear to be characteristic for cells with optimal intracellular formate concentration during mixed-acid fermentation.

Finally, measurement of pH_i_ for all three strains after 12 h of cultivation in M9-glucose medium at pH 8 revealed that for the WT and for FocAT91A, the values were 6.7 and 6.81, respectively (Table S2). The pH_o_ profiles in the culture medium during cultivation were also similar for both strains, but with a pH_o_ = 5.3 (phase E), the ΔpH was 1.39 for WT and 1.53 for FocAT91A (Table S2). In the case of strain FocAH209N, which continuously translocates formic acid out of the cell (18), the pH_o_ after 12 h of cultivation was 5.79 and the pH_i_ was 6.92, indicating a ΔpH = 1.13 (Table S2).

### Intracellular formate levels are severely restricted in FocAH209N (DH4200)

Overall differences in intracellular formate levels in *E. coli* cells can be monitored by using a formate reporter in which the *lacZ* gene is fused to the FhlA- and formate-dependent promoter of the *fdhF* gene (18, 23). Previous studies have reported that in FocAH209N during glucose fermentation, β-galactosidase enzyme activity cannot be measured because the formate generated by PflB is so efficiently exported from the cell by the FocA_N209_ variant that the formate concentration is below the *K*_d_ of FhlA for formate (14). Similarly, strain FocAT91A has considerably higher levels of intracellular formate than the WT (22), because formate accumulates in the cytoplasm of FocAT91A due to impaired FocA_A91_-dependent efflux of formic acid, and this leads to high β-galactosidase enzyme activity (22).

Analysis of β-galactosidase enzyme activity during anaerobic cultivation of the three strains in M9-glucose medium, at pH 8, revealed an activity for WT in phases D and E (see Fig. 1) of 80 and 50 Miller units, while in the same phases of cultivation, FocAT91A showed activities of 730 and 100 Miller units, respectively (Fig. S4). The activity of FocAT91A was therefore nearly 10-fold higher in early stationary phase D compared with that in the parent, reflecting a presumptive 10-fold difference in intracellular formate levels. No β-galactosidase enzyme activity could be measured in any of the phases of cultivation of FocAH209N under these conditions (data not shown).

**Fig 4:**
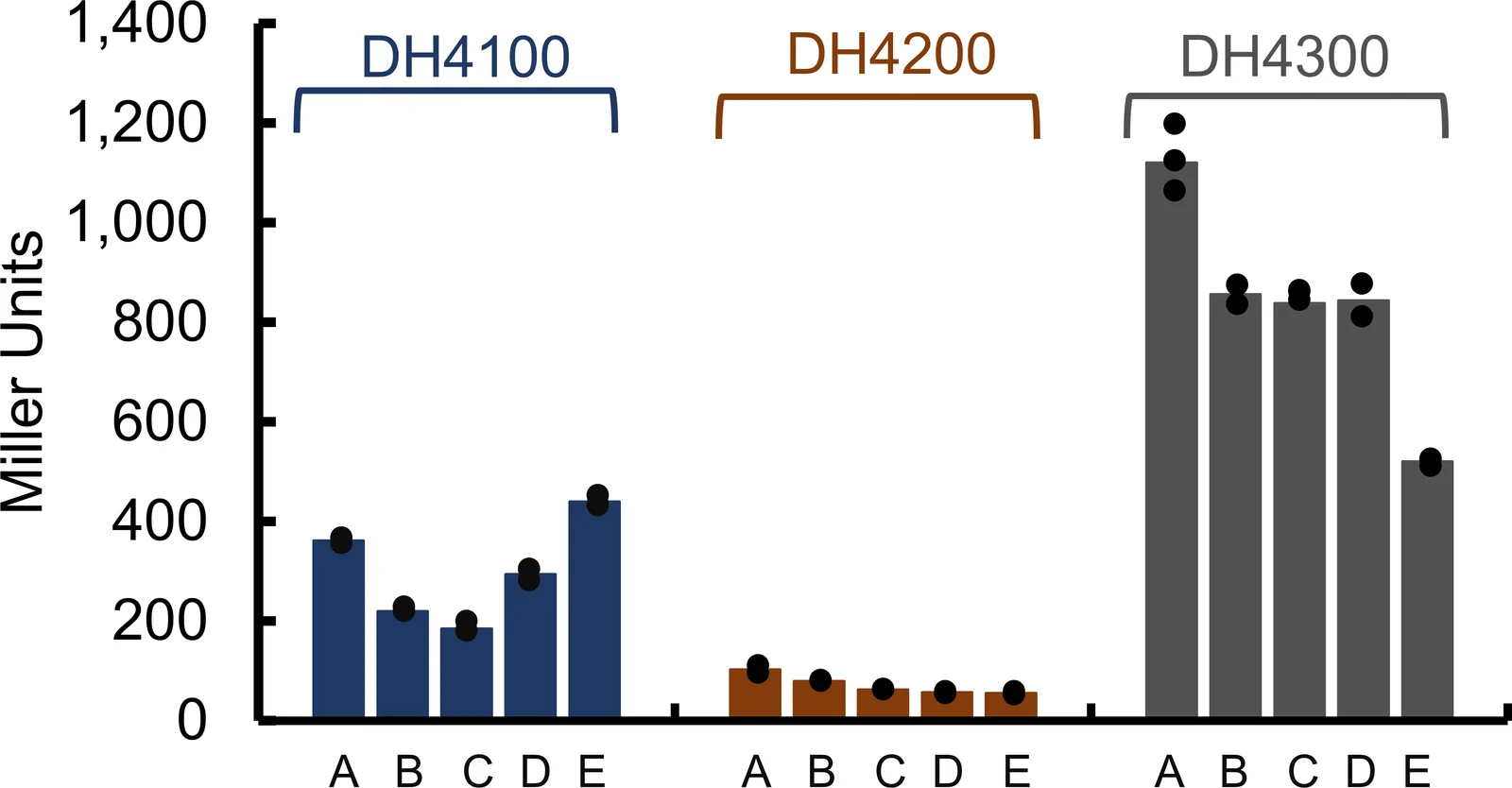
Formate-dependent expression of *fdhF_P_::lacZ* during fermentative growth in the presence of 50 mM sodium bicarbonate. β-Galactosidase enzyme activity of WT (DH4100—dark blue histograms), FocAH209N (DH4200—dark orange histograms), and FocAT91A (DH4300—dark gray histograms) after anaerobic cultivation in M9-glucose medium, pH 7, supplemented with 50 mM sodium bicarbonate. Parameters were determined as described in the legend to Fig. 2 and as depicted in Fig. 1. Data represent values obtained with three biological replicates for each strain with each data point shown.

Cultivation of the three strains in the presence of 50 mM bicarbonate resulted in an increase in the β-galactosidase enzyme activity, relative to growth without bicarbonate, to ~400 Miller units measured in stationary-phase cells (phase E) of the WT (Fig. 4; Statistics Table S4). Very low levels of enzyme activity could be measured for FocAH209N (~ 50 Miller units), while FocAT91A had β-galactosidase enzyme activity values of around 800 units during exponential growth (phases B and C), decreasing to 500 Miller units in stationary-phase cells (phase E). These data indicate that the likely reason for the inability of 50 mM bicarbonate to restore growth and ATP levels to strain FocAH209N to those of the WT was due to low intracellular formate levels. In support of this conclusion, 5.3 μmol, 0.02 μmol, and 4.28 μmol of accumulated H_2_ (Table S3) were detected in each 0.2 ml of headspace gas after cultivation of WT, FocAH209 (formate efflux mutant) and FocAT91A (mutant defective in formate efflux), respectively, and which had been grown for 12 h in M9-glucose medium supplemented with 50 bicarbonate. This result confirms that FHL-1 activity was not restored to wild-type levels for strain FocAH209N after cultivation in the presence of bicarbonate.

Complementation of the mutant phenotype of strain DH4200 with the wild-type *focA* gene on plasmid pfocA (18) proved only partially successful due to the dominance exhibited by FocA_N209_ over the native FocA protein (24). While wild-type anaerobic growth was not recovered in the plasmid-complemented FocAH209N strain, H_2_ production was restored to 1.61 μmol of accumulated H_2_ per 0.2 mL (Table S3) of headspace gas after cultivation of FocAH209N/pfocA in M9-glucose medium pH 7 for 12 h. Strain FocAH209N without the plasmid accumulated 0.54 μmol H_2_ per 0.2 mL of headspace gas (Table S3) under the same conditions, which was approximately 12% of the amount produced by WT.

### Growth in the presence of bicarbonate increased extracellular formate levels in stationary phase cultures of FocAH209N (DH4200)

**Fig 5:**
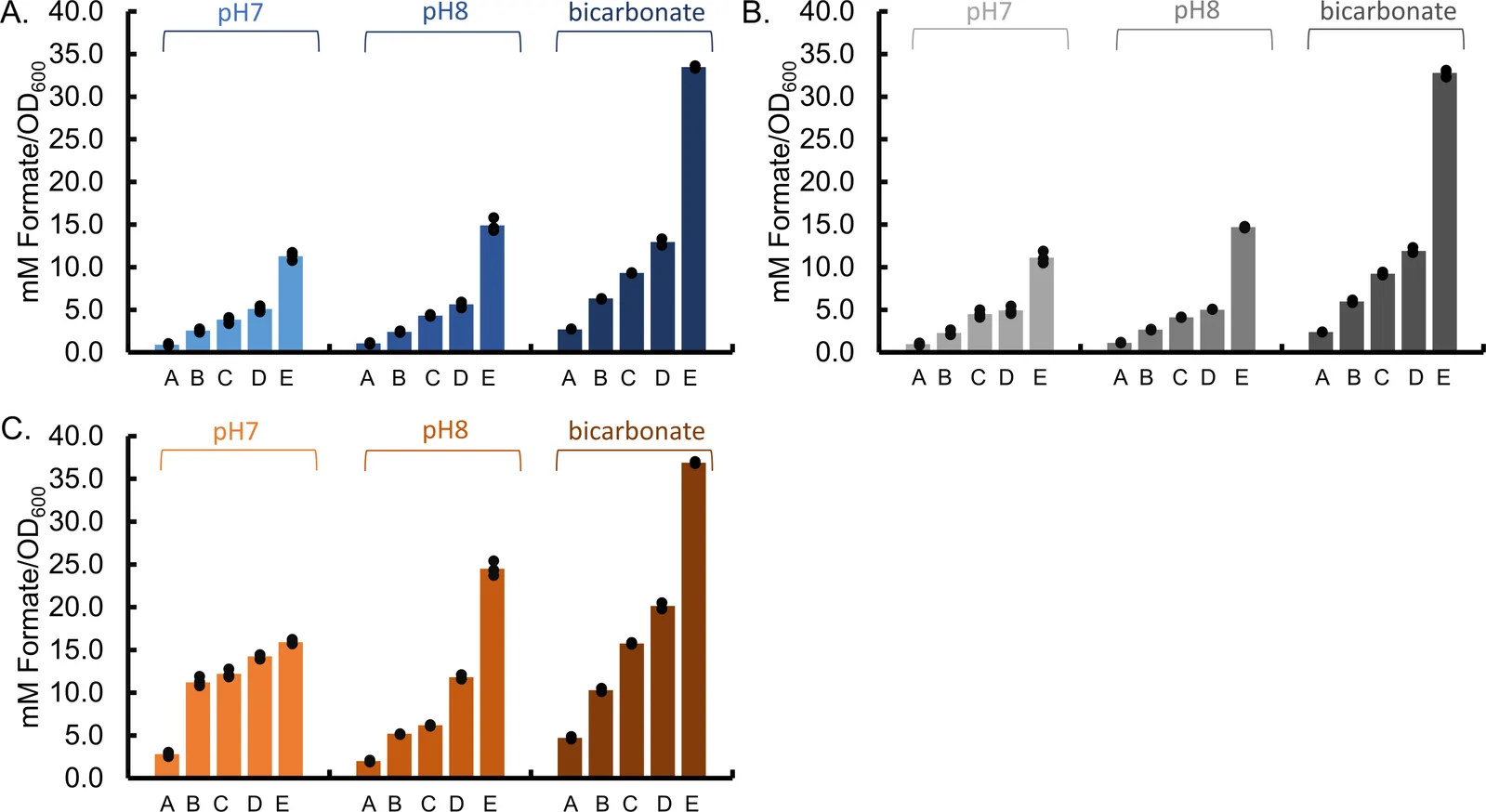
Increased extracellular formate concentrations produced by strains cultivated with bicarbonate. The formate concentrations are shown for WT (DH4100, **A**), FocAH209N (DH4200, **B**), and FocAT91A (DH4300, **C**) measured in the culture medium during growth under the indicated conditions, depicted above each set of histograms, and exactly as described in the legend to Fig. 2. Parameters were determined as described in the legend to Fig. 2. Data points are shown for three biological replicates.

Measurement of the extracellular formate concentration after these growth experiments revealed that formate levels did not differ significantly when the WT and strain FocAT91A were cultivated at pH 7 or at pH 8 (Fig. 5A and B). A significant difference in extracellular formate levels was, however, observed after cultivation of both strains in the presence of 50 mM bicarbonate. Extracellular formate levels were nearly 3-fold higher for both strains in stationary phase cultures supplemented with 50 mM bicarbonate when compared with the same phase for pH 7 cultures without bicarbonate supplementation (Fig. 5; Statistics Table S5).

Growth of FocAH209N in M9-glucose minimal medium at pH 7 showed formate concentrations to be similar to those determined previously (18), and these were between 10 and 15 mM formate OD_600_^−1^ in phases B through E (Fig. 5C). Notably, however, during growth at pH 8, the profile of formate production mimicked that of the WT, albeit the measured levels of formate were approximately 30%–40% higher (Fig. 5C). During cultivation of FocAH209N in the presence of 50 mM bicarbonate, the concentration of formate in the culture medium of stationary-phase cells was similar to that determined for both WT and FocAT91A (compare Fig. 5A and B); however, during the later stages of exponential growth, and upon transition into the stationary phase (phases B, C, and D, respectively), the formate concentration in the culture medium of strain FocAH209N was significantly higher (~30%) than that measured for both of the other strains (Fig. 5C; Statistics Table S5).

### Strain FocAH209N (DH4200) shows a major change in its fermentation product spectrum

**Fig 6:**
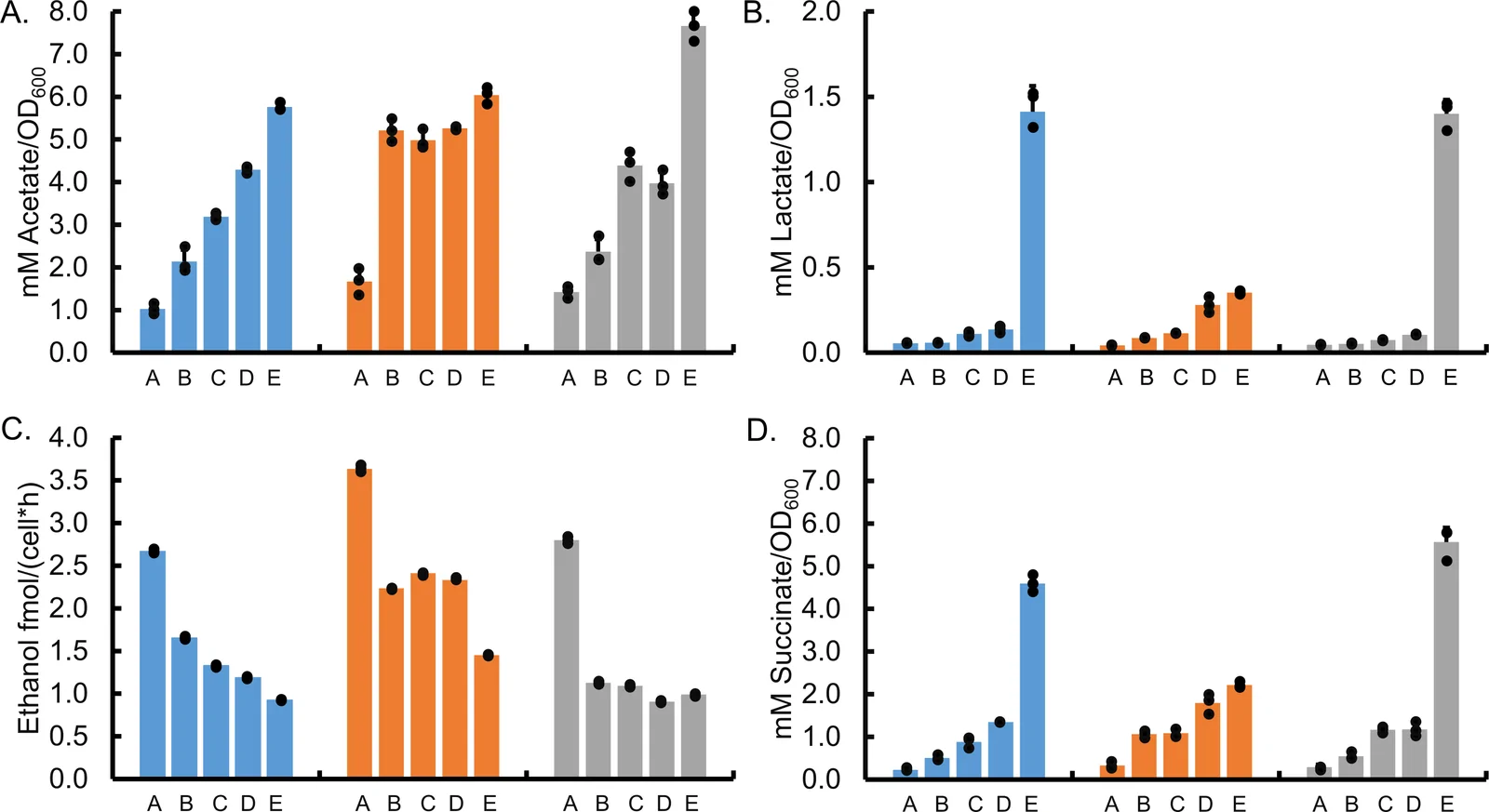
Fermentation product profiles of DH4100 (WT), DH4200 (FocAH209N), and DH4300 (FocAT91A) during glucose fermentation. The concentrations of acetate (**A**), lactate (**B**), ethanol (**C**), and succinate (**D**) are shown for each strain during cultivation in M9-glucose minimal medium, pH 7. Parameters were determined as described in the legend to Fig. 2. Data points are shown for three biological replicates.

The continuous efflux of formic acid by strain FocAH209N during fermentative growth suggested that other fermentation products might show altered levels and could exacerbate the impaired growth-phenotype of the strain. Therefore, the levels of acetate, lactate, ethanol, and succinate were determined for WT, FocAH209N, and FocAT91A during cultivation in M9-glucose medium (Fig. 6). Generally, the levels of ethanol, lactate, and succinate throughout growth were not statistically different for the WT and for FocAT91A, while acetate accumulated to slightly higher levels in FocAT91A (Fig. 6; see Statistics Table S6). Lactate and succinate accumulated to 1.5, and 4.5–5.0 mM OD_600_^−1^, respectively, in the stationary-phase culture medium of both strains. In contrast, strain FocAH209N produced only 0.4 mM OD_600_^−1^ lactate (Fig. 6B), ~6 mM OD_600_^−1^ acetate (Fig. 6A), and produced succinate to approximately 2 mM OD_600_^−1^ (Fig. 6D) in the stationary-phase culture medium.

Ethanol was present at a high relative level in the lag phase, but the level decreased similarly for WT and FocAT91A once growth initiated (Fig. 6C). However, the culture medium of FocAH209N contained significantly more ethanol than that of either of the other two strains (Statistics Table S6). These results indicate that strain FocAH209N produced significantly more ethanol (~50%), presumably as a means of reoxidizing the NADH that accumulated during glycolysis, because insufficient levels of lactate and succinate were produced by the strain.

### Bicarbonate supplementation increases organic acid levels produced by FocAH209N (DH4200)

**Fig 7:**
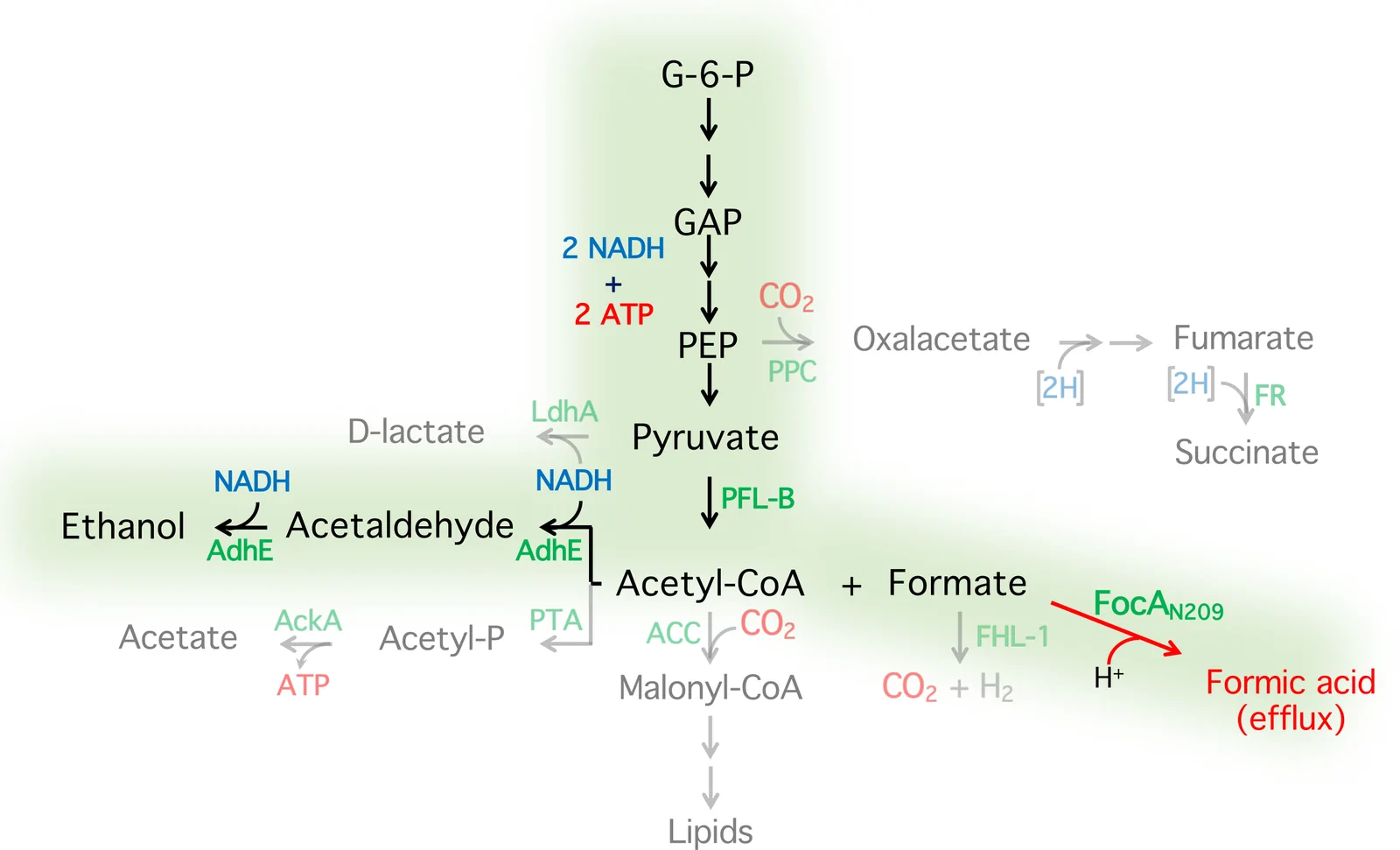
Key mixed-acid fermentation products of strain DH4200 (FocAH209N). A schematic representation of key pathways and some enzymes during mixed-acid fermentation are shown, with the additional route to lipid synthesis also depicted. The green background highlights the main metabolic routes delivering the fermentation products of FocAH209N, which synthesizes FocA_N209_. The pathways depicted in gray highlight which products are typically generated and where limitations are observed in FocAH209N, especially regarding CO_2_/bicarbonate involvement. Abbreviations: ACC, acetyl-CoA carboxylase; AckA, acetate kinase; AdhE, alcohol dehydrogenase/acetaldehyde dehydrogenase; FHL-1, formate hydrogenlyase; FR, furmarate reductase; LdhA, D-lactate dehydrogenase; PflB, pyruvate formate-lyase; PPC, phosphoenolpyruvate carboxylase; PTA, phosphotransacetylase.

To determine the effect of bicarbonate supplementation on the fermentation product spectrum of FocAH209N, all three strains were cultivated in M9-glucose medium containing 50 mM bicarbonate and the concentrations of the fermentation products in the growth medium were determined throughout all phases of growth (Fig. S5; Statistics Table S7). Bicarbonate supplementation had the general effect of increasing the relative concentrations of acetate and succinate for all three strains, while lactate and ethanol levels were lowered compared to when the strains were grown without bicarbonate supplementation (compare Fig. 6 and Fig. S5). That this effect was due to altered pH_o_ could be excluded by repeating the experiment in M9-glucose minimal medium, pH 8 (Fig. S6; Statistics Table S8), and suggests that the availability of bicarbonate increased the carboxylation of PEP, generating malate and fumarate as an alternative route of achieving redox balance (see Fig. 7).

While bicarbonate did not restore the fermentation product spectrum of FocAH209N to that determined for the WT, the levels of acetate (Fig. S5A), lactate (Fig. S5B), and succinate (Fig. S5D) were nonetheless increased compared with the levels measured after cultivation without bicarbonate-supplementation (compare Fig. 6); this was particularly evident in stationary-phase cultures. The level of ethanol was decreased by approximately 50% (Fig. S5C) relative to the level in the culture medium after growth of the strain in the absence of bicarbonate. However, the level did not attain the level determined for the WT (see Fig. 6C).

## DISCUSSION

The aberrant anaerobic phenotypes in stationary-phase survival of *E. coli* mutants lacking FHL-1 appear to be due directly to insufficient amounts of CO_2_ generated in the cytoplasm and to reduced levels of ATP (9). Similar effects likely cause the severe growth phenotype observed in *E. coli* strain FocAH209N (DH4200), which synthesizes the FocA_N209_ variant that excretes formic acid more efficiently than importing it (18). Due to the very low intracellular levels of formate in FocAH209N, the FHL-1 complex is not synthesized to wild-type levels. The consequence is that too little formate is disproportionated to CO_2_ (and H_2_ to maintain redox control) to satisfy the cell’s requirement for key carboxylation reactions. Because the FocA_N209_ variant made by FocAH209N is also incapable of efficient re-import of formate (18, 19), especially in stationary phase cells, this exacerbates the phenotypic deficiency. Control experiments with FocAT91A (DH4300), which synthesizes a different FocA_A91_ variant that has a defect in formic acid efflux, and thus accumulates formate intracellularly (22), supported this conclusion. Indeed, FocAT91A grew slightly better than even the WT, which was likely the result of a constant and optimal pH_i_ of 6.8 throughout all phases of cultivation, along with the ready availability of intracellular formate.

Supplying fermenting cultures of FocAH209N with high concentrations of sodium bicarbonate rescued the phenotypic deficiency of the strain in large part, but not completely. This contrasts the observed complete phenotypic recovery of FHL-1-negative mutants by supplying bicarbonate (9). Analysis of pH_i_, ATP, and F_1_F_o_-ATPase activity levels, and the fermentation product spectrum of FocAH209N cultivated with and without bicarbonate, revealed that the incomplete phenotypic restoration of growth by bicarbonate was likely due to two effects: a significant and continuous increase in the ATP-hydrolyzing activity of the F_1_F_o_-ATPase; and a shift in metabolism towards more ethanol production (Fig. 7), the latter having potentially detrimental effects on membrane integrity (25). The rapid and continuous efflux of formic acid through the FocA_N209_ channel in FocAH209N causes a constant limitation of the anion, formate. As PflB has a high affinity for pyruvate and a high turnover rate (1, 26, 27), pyruvate is very efficiently cleaved into acetyl-CoA and formate as the cell attempts to supply formate for the FHL-1 reaction. Thus, PflB outcompetes LdhA for the substrate pyruvate. This explains why there is no increase in lactate concentration in the growth medium of stationary-phase FocAH209N cultures, but which typically is found in the stationary-phase culture medium of the parental strain (see Fig. 6). Succinate is also present at only low levels in the culture medium of FocAH209N because bicarbonate, which is generated from CO_2_ by carbonic anhydrase, particularly in stationary-phase cells (3), is unavailable as a co-substrate for the PEP carboxylase reaction (Fig. 7). The fact that LdhA cannot be used to help re-oxidize the NADH generated during glycolysis means that a greater proportion of acetyl-CoA has to be sacrificed in stationary-phase cells of FocAH209N to re-oxidize NADH, catalyzed by AdhE. This also means that proportionately less acetyl-CoA is available for ATP generation by the combined activities of the phosphotransacetylase and acetate kinase branch of fermentation. This also explains, in part, why bicarbonate fails to restore a wild-type growth phenotype to strain FocAH209N (see Fig. 1B).

Measurement of ATP levels in stationary-phase cells of FocAH209N after cultivation in the presence of bicarbonate revealed a threefold reduction compared with WT from an early stage in cultivation (see Fig. 2). Yet, the final cell density attained by these cultures of FocAH209N grown in the presence of bicarbonate was only approximately 20% lower than that attained by similar cultures of the WT. Lack of an adequate bicarbonate supply in FocAH209N grown in M9-glucose medium is, thus, a key factor in the lower cell density attained by these cells, and we suggest that it is due to limitations in lipid biosynthesis (Fig. 7). Restricted lipid biosynthesis results in a destabilization of the cytoplasmic membrane (3, 4), and this will be further compounded by the increased ethanol production by FocAH209N (25).

The early entry of FocAH209N into stationary phase correlated strongly with the low ATP levels in the cells and was caused by enhanced F_1_F_o_-ATPase-catalyzed ATP hydrolysis. A similar correlation was also shown for mutants lacking FHL-1, but ATP hydrolysis began at a later stage during cultivation (9). The premature activation of the ATP-hydrolyzing activity of F_1_F_o_-ATPase in FocAH209N strongly suggests a link with membrane destabilization caused by the combined deficiency in bicarbonate for the acetyl-CoA carboxylase and PEP carboxylase reactions and enhanced ethanol production. Membrane destabilization may well represent one signal stimulating the activation of ATP hydrolysis by F_1_F_o_-ATPase (28, 29). However, although the addition of high levels of exogenous bicarbonate caused an increase in the cell density and delayed the transition into stationary phase (see Fig. 1B), this did not result in an increase in ATP levels once stationary phase was reached by FocAH209N cells (see Fig. 2B). This was also partially reflected in the pH_i_ of these cells, which during cultivation in the presence of bicarbonate did not decrease below 6.9. These findings strongly suggest that at least one further signal is sensed by the F_1_F_o_-ATPase to maintain its ATP-hydrolyzing activity in stationary phase cells of FocAH209N, even when growing with exogenous bicarbonate. Whether the approximately 40% increase in ethanol production, relative to the WT strain, in stationary phase cultures of FocAH209N was sufficient to induce activity of the ATPase remains to be tested. It nevertheless seems clear that a consequence of enhanced ATP hydrolysis in FocAH209N cells helped to maintain the pmf near 200 mV. Cultivation of FocAH209N cells in M9-glucose medium resulted in a pH_i_ that was maintained at approximately 7 throughout growth and into the stationary phase. This pH_i_ was maintained by proton efflux through the F_1_F_o_-ATPase to conserve pmf, but presumably also to enhance the trapping inside the cell of any available CO_2_ as the bicarbonate anion (3).

The demonstration that FocAT91A, which accumulates formate intracellularly, maintained a pHi of 6.8 throughout all growth phases supports the proposal (9) that this pH is optimal for balanced mixed-acid fermentation. Moreover, this contention is further supported by the slightly better growth of FocAT91A compared with its parent.

In contrast to FocAH209N, FHL-1-negative strains have a high intracellular formate level (9), and this formate is not lost rapidly from the cell as it is in FocAH209N (18). Low formate potentially also influences the early entry of cells into stationary phase, as formate is required for deoxyribonucleotide biosynthesis in microorganisms that use PflB-like enzymes to cleave pyruvate (5, 30). Thus, a carefully balanced level of intracellular formate, attained through controlled PflB activity, bidirectional formate translocation, and formate-induced FHL-1 synthesis and activity, ensures optimal conditions for anaerobic fermentative growth. Any imbalance in formate levels, whether through impaired PflB activity (13), aberrant unidirectional efflux by FocA, or a lack in FHL-1 activity, severely impacts mixed-acid fermentation. A limitation in intracellular formate has a severe knock-on effect on pH_i_, ATP levels and membrane stability (Fig. 7).

Finally, the results of this study, and other recent work (9), indicate that even apparently minor metabolic changes that affect intracellular CO_2_ levels can have significant consequences for the cell’s survival, particularly in stationary phase, and how it reprograms its metabolic pathways accordingly. A clear elucidation of the direct signals that are sensed to elicit the metabolic changes in ATP and pH_i_ levels in response to CO_2_ limitation is an important challenge for future studies. Our current findings point toward maintenance of membrane structure and integrity as being cardinal in this process, with bicarbonate and CO_2_ as the potential primary signal molecules and acetyl-CoA carboxylase (31), carbonic anhydrase (3, 32), and possibly the F_1_F_o_-ATPase (29), as sensors.

## MATERIALS AND METHODS

### Strains and growth conditions

The parental strain of *E. coli* used in this study was DH4100 (23) and is referred to as WT throughout the manuscript. DH4100 is like MC4100 (F^-^ *araD* Δ(*argF lac*) *U 169 ptsF25 deoC1 relA1 fblB530 rpsL 150 λ^-^*; 33) but has a single-copy of a *λfdhF_P_::lacZ* fusion in its genome. Strains DH4200 and DH4300 are isogenic derivatives of DH4100, whereby DH4200 has codon 209 of the *focA* gene changed to AAC (His to Asn exchange) (18), and DH4300 has codon 91 of the *focA* gene changed to GCT (Thr to Ala exchange) (22). DH4200 is referred to as FocAH209N and DH4300 as FocAT91A throughout the text.

The plasmid used was pFocA (*focA*^+^) (18).

All strains were grown anaerobically in liquid M9-minimal medium, pH 7.0, with 0.8% (wt/vol) glucose as a carbon source, and at 37°C, exactly as described (34). Occasionally, growth studies were done at pH 8.0, where indicated, but otherwise, medium content was the same. Where indicated, the medium was supplemented with 50 mM sodium bicarbonate (Carl Roth, Karlsruhe, Germany). Anaerobic growth analysis was carried out in 96-well microtiter plates, as previously described (34), but was monitored using a Tecan Spark Microplate Reader (Männedorf, Switzerland). Kanamycin was supplemented to the growth medium at a final concentration of 50 μg mL^−1^ for the selection of strains, and when required, ampicillin was added at a final concentration of 100 μg mL^−1^ for plasmid selection.

### Determination of intracellular ATP levels

Intracellular ATP levels were determined using the luciferase-based ATP-Bioluminescence-Assay-Kit CLS II (Roche Diagnostics, Basel, Switzerland) and whole cells sampled at different stages of growth. This was done exactly as recently described (9).

### Determination of extracellular and intracellular pH

Extracellular pH_o_ was measured in the culture medium using a 765 Calimatic pH meter (Knick Elektronische Messgeräte GmbH & Co. KG, Germany) equipped with a WTW pH SenTix Mic microelectrode (Fischer Scientific GmbH, Germany), as described (9). Intracellular pHi was determined using 2',7'-bis(2-carboxyethyl)−5-(and 6)-carboxyfluorescein-acetoxymethyl-ester (BCECF-AM, Thermo Fisher Scientific, USA) following the methodology detailed in Erdmann et al. (9). Cell suspensions (1 mL) were sampled at the indicated time-points during growth.

Fluorescence was measured using black-walled 96-well microtiter plates in a Tecan Spark plate-reader at excitation wavelengths of 440 and 490 nm and an emission wavlength of 505 nm over a period of 30 min at 37°C. Experiments were performed using three biological replicates, with each replicate performed in duplicate. Data are presented as standard deviation of the mean.

### Measurement of organic acids and ethanol during fermentative growth

Determination of acetate, formate, lactate, and succinate concentrations in the growth medium was done by high performance liquid chromatography (HPLC), with monitoring at 210 nm, exactly as described (18). The concentration of each organic acid was calculated with reference to the optical density (OD_600 nm_).

Ethanol concentration was measured by placing 1.5 mL of cell culture in an Eppendorf tube followed by centrifuging for 7 min at 14,000 rpm in a bench-top centrifuge. Subsequently, 0.8 mL of the cell-free supernatant was transferred to a 2 mL glass tube to which 160 μL of phosphoric acid (4.25%, vol/vol) was added. This acidification step (0.7%, vol/vol, final percentage concentration) prevented any further metabolic activity by significantly reducing the pH to 2 and the vapor pressure, which resulted in a sharper ethanol peak. The glass tube was sealed with a rubber stopper that allowed automatic gas-sampling using a GC2010 Plus gas-chromatograph (Shimadzu, Duisburg, Germany). Ethanol was detected using a flame ionization detector with N_2_ as carrier gas.

Ethanol concentration was determined and presented as fmol cell^−1^ h^−1^. The experiments were performed with three biological replicates, each with three technical replicates, and data are presented as standard deviation of the mean.

### Monitoring changes in intracellular levels of formate

Changes in intracellular formate levels were determined indirectly by measuring the β-galactosidase enzyme activity, represented in Miller units (35), exactly as previously described (18). This method does not measure changes in concentration of formate but monitors whether formate is present, reflected in induction of β-galactosidase enzyme activity, and when clear increases and decreases are observed, as explained recently (9).

### Measurement of membrane potential and determination of pmf

Membrane potential (Δ*Ψ*) was determined as described (36) from the steady-state distribution of tetraphenylphosphonium (TPP^+^) between bacterial cells and the external medium. A cell volume of 0.785–0.821 × 10⁻¹² was assumed for cells grown between pH 6.5 and 7.5 (36). Extracellular TPP^+^ concentration was monitored with a TPP^+^-selective electrode in 150 mM Tris–HCl buffer containing 1 μM TPP^+^. Non-specific TPP^+^ adsorption was corrected using boiled cells. For cells grown at pH 6.5–7.5, we use a volume of 0.785–0.821 × 10⁻¹² (calculated based on cell length and width).

Proton motive force (pmf) was calculated for a temperature of 37°C using the formula: Δp = ΔΨ – 60 mV × ΔpH.

### Measurement of ATPase enzyme activity

Total ATPase and the contribution to this activity made by the F_1_F_O_-ATPase after incubation with *N,N'*-dicyclohexylcarbodiimide, was determined in membrane vesicles, exactly as described (9, 37). Protein concentration in the membrane vesicles was determined by the Lowry method using bovine serum albumin as standard (38).

### Measurement of H_2_ and CO_2_ production via gas chromatography

Cultivation of the strains for determination of H_2_ and CO_2_ was done in M9-glucose minimal medium in 15 mL Hungate tubes filled with 5 mL of medium (10 mL head-space), under a N_2_ atmosphere (33). Accumulated H_2_ and CO_2_ were measured in the head-space of cultures grown anaerobically for 12 h and at 37°C (9, 18). Two independent experiments were done, and the amounts of H_2_ or CO_2_ were calculated with reference to the volume of gas-headspace.

### Statistical analyses

Statistical analysis was performed either using variance analysis employing two-way ANOVA followed by Tukey’s multiple comparisons test, of by performing a three-way ANOVA multiple comparison analysis using JASP (Jeffrey’s Amazing Statistics Program).

## SUPPLEMENTAL MATERIAL

Supplemental material jb.00356-26-s0001.pdf Fig. S1 to S6; Tables S1 to S3; Statistic Tables S1 to S8.

ASM does not own the copyrights to Supplemental Material that may be linked to, or accessed through, an article. The authors have granted ASM a non-exclusive, world-wide license to publish the Supplemental Material files. Please contact the corresponding author directly for reuse.
