# Supplementary material for: Importance of balanced intracellular formate levels for pHi and CO2 homeostases during mixed-acid fermentation
Source: J Bacteriol. 2026 Sep 3;208(10):e00356-26. doi: 10.1128/jb.00356-26 (PMC13689563; doi:10.1128/jb.00356-26)
Supplement: Supplementary file 1 [file jb.00356-26-s0001.pdf]

Supplementary Material

Importance of balanced intracellular formate levels for pH_i and CO_2 homeostases during mixed-acid fermentation

Christopher Erdmann[a], Liana Vanyan[b], Karen Trchounian[b], and R. Gary Sawers[a]#

[a] Institute for Microbiology, Martin Luther University Halle-Wittenberg, Halle (Saale), Germany

[b] Department of Biochemistry, Microbiology and Biotechnology, Faculty of Biology, Yerevan State University, Yerevan, Armenia

Running head: Formate-based control of mixed-acid fermentation

# Address correspondence to R. Gary Sawers, gary.sawers@mikrobiologie.uni-halle.de

Table S1. Proton gradient measurements during fermentation in membrane vesicles

Strain	6h[a]			12h[a]		
	ΔpH	ΔΨ (mV)	pmf (mV)	ΔpH	ΔΨ (mV)	pmf (mV)
DH4100	0.2	-99 ± 5.7	-111	1.5	-131 ± 3.2	-221
DH4200	0.8	-96 ± 11.6	-144	1.3	-135 ± 18	-213
DH4300	0.15	-93 ± 8.5	-102	1.4	-181 ± 19 ***	-265

[a] Timepoints during anaerobic growth in M9-glucose minimal medium of sample analysis.

*Values are presented as mean ± SD (n = 3). Statistical analysis was performed using two-way ANOVA followed by Tukey's multiple comparisons test. Asterisks denote significant differences compared with the corresponding wild-type strain (DH4100) at the same growth phase (***P < 0.001); values without asterisks were not significantly different.

Table S2. Measurement of ΔpH in stationary phase after 12h cultivation under different fermentation conditions.

Strain and growth condition	pH_i	pH_o	ΔpH
DH4100, M9-glucose, pH 7	6.68	5.3	1.38
DH4100, M9-glucose, pH 8	6.7	5.31	1.39
DH4100, M9-glucose, pH 7 + 50 mM bicarbonate	6.8	6.12	0.68
DH4200, M9-glucose, pH 7	7.05	5.7	1.35
DH4200, M9-glucose, pH 8	6.92	5.79	1.13
DH4200, M9-glucose, pH 7 + 50 mM bicarbonate	6.92	6.28	0.64
DH4300, M9-glucose, pH 7	6.8	5.4	1.4
DH4300, M9-glucose, pH 8	6.81	5.28	1.53
DH4300, M9-glucose, pH 7 + 50 mM bicarbonate	6.82	6.08	0.74

Table S3. Measurement of H_2 accumulation in the gas-phase of stationary phase cultures (12 h) after growth in M9-glucose minimal ± 50 mM sodium bicarbonate.

Strain[a]	H_2 accumulation (μmol 0.2 ml^{-1})	H_2 accumulation (μmol 0.2 ml^{-1}) + 50 mM bicarbonate
WT (DH4100)	4.69 ± 0.05	5.3 ± 0.05
FocAH209N (DH4200)	0.54 ± 0.08	0.02 ± 0.007
FocAH209N + pfocA[b]	1.61 ± 0.01	n.d.[c]
FocAT91A (DH4300)	4.78 ± 0.07	4.28 ± 0.15

[a], experiments were performed minimally twice (biological replicates) and standard deviation of the mean is shown; [b], OD600nm = 0.45; [c], not determined

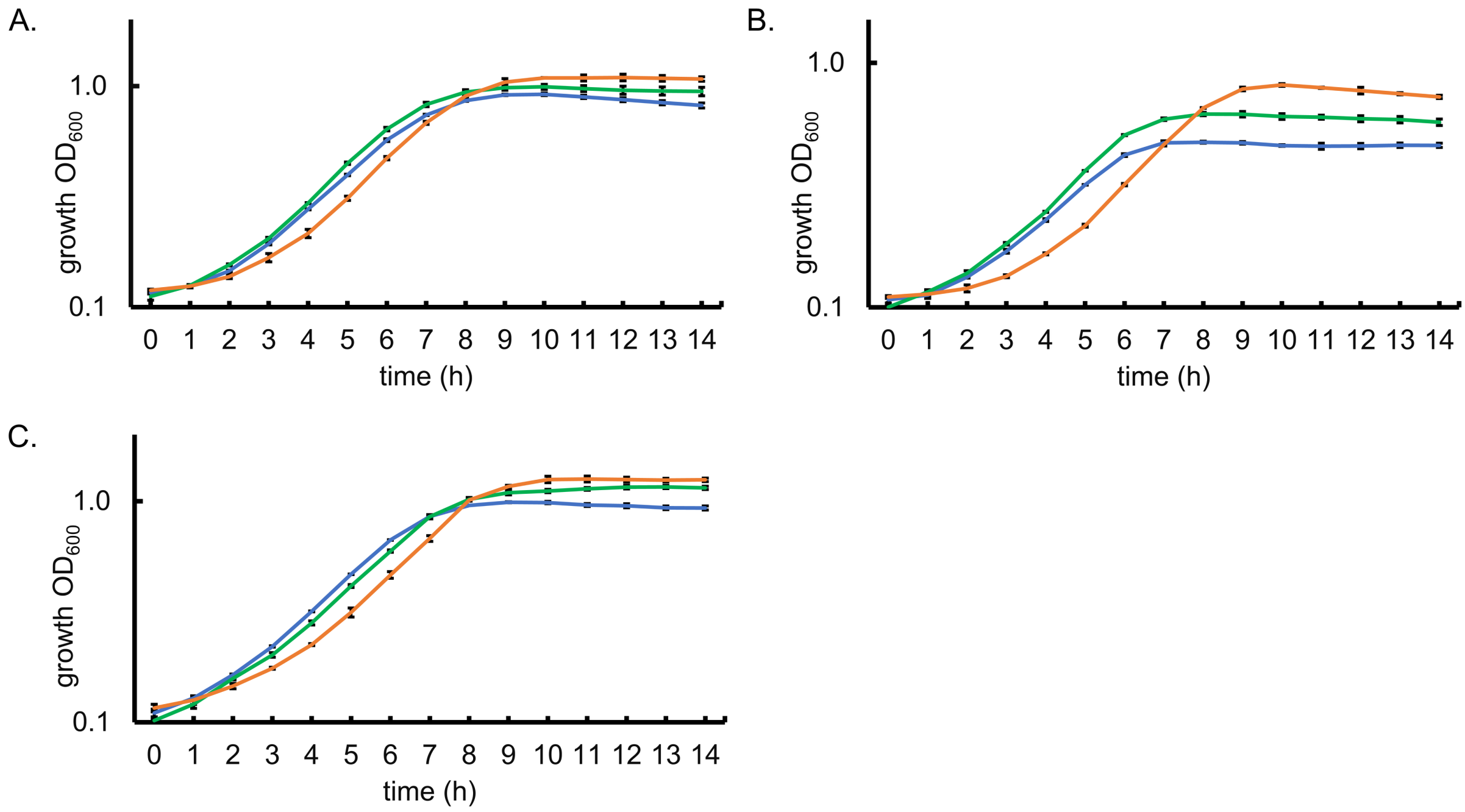

Figure S1. Growth during glucose fermentation of a mutant continuously exporting formic acid is severely restricted. Growth curves of the strains shown in Fig.1 of the main text represented in semi-logarithmic formate. The strains analyzed include DH4100 (parental strain, panel A); DH4200 (mutant synthesizing $FocA_{N209}$, panel B); DH4300 (mutant synthesizing $FocA_{A91}$, panel C). Each strain was grown anaerobically in M9-glucose medium, pH 7 (blue line), in M9-glucose medium, pH 8 (green line), or in M9-glucose medium, pH 7 supplemented with 50 mM sodium bicarbonate (orange line). The growth experiments were performed with three biological and three technical replicates for each strain. Standard error of the mean is shown for each data point.

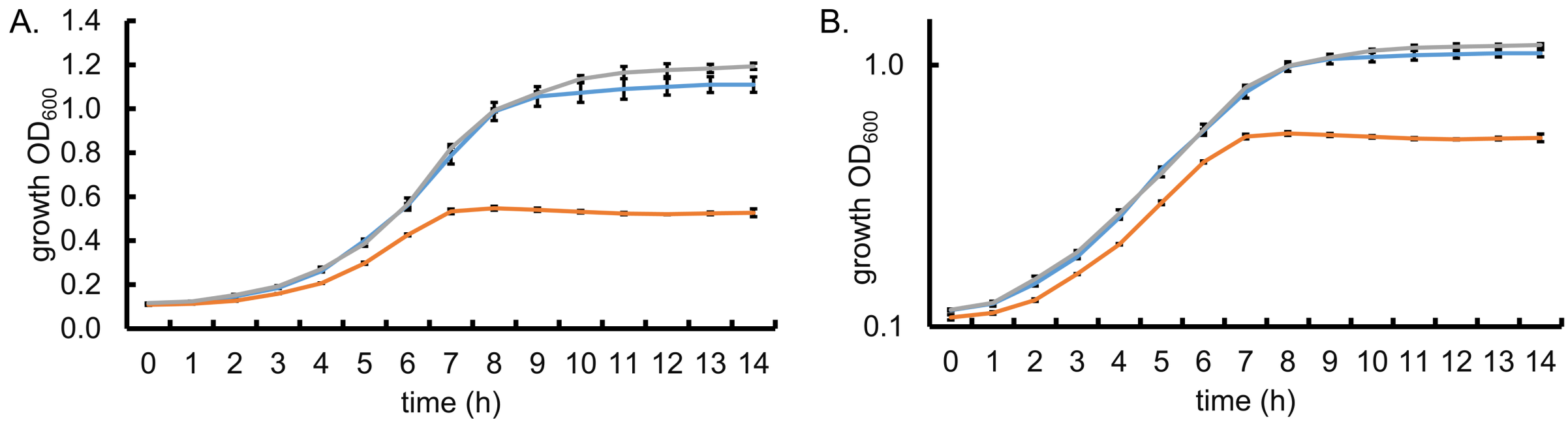

Figure S2. Impact of 10 mM sodium bicarbonate on fermentative growth during glucose fermentation. The parental strain, DH4100 (blue curve), and the mutants synthesizing FocA amino acid variants $FocA_{N209}$ (DH4200, orange curve) and $FocA_{A91}$ (DH3400, gray curve) were grown in M9-glucose medium supplemented with 10 mM soidium bicarbonate. Growth curves are depicted in linear (panel A) and semi-logarithmic formats (panel B). The growth experiments were performed with three biological and three technical replicates for each strain. Standard error of the mean is shown for each data point.

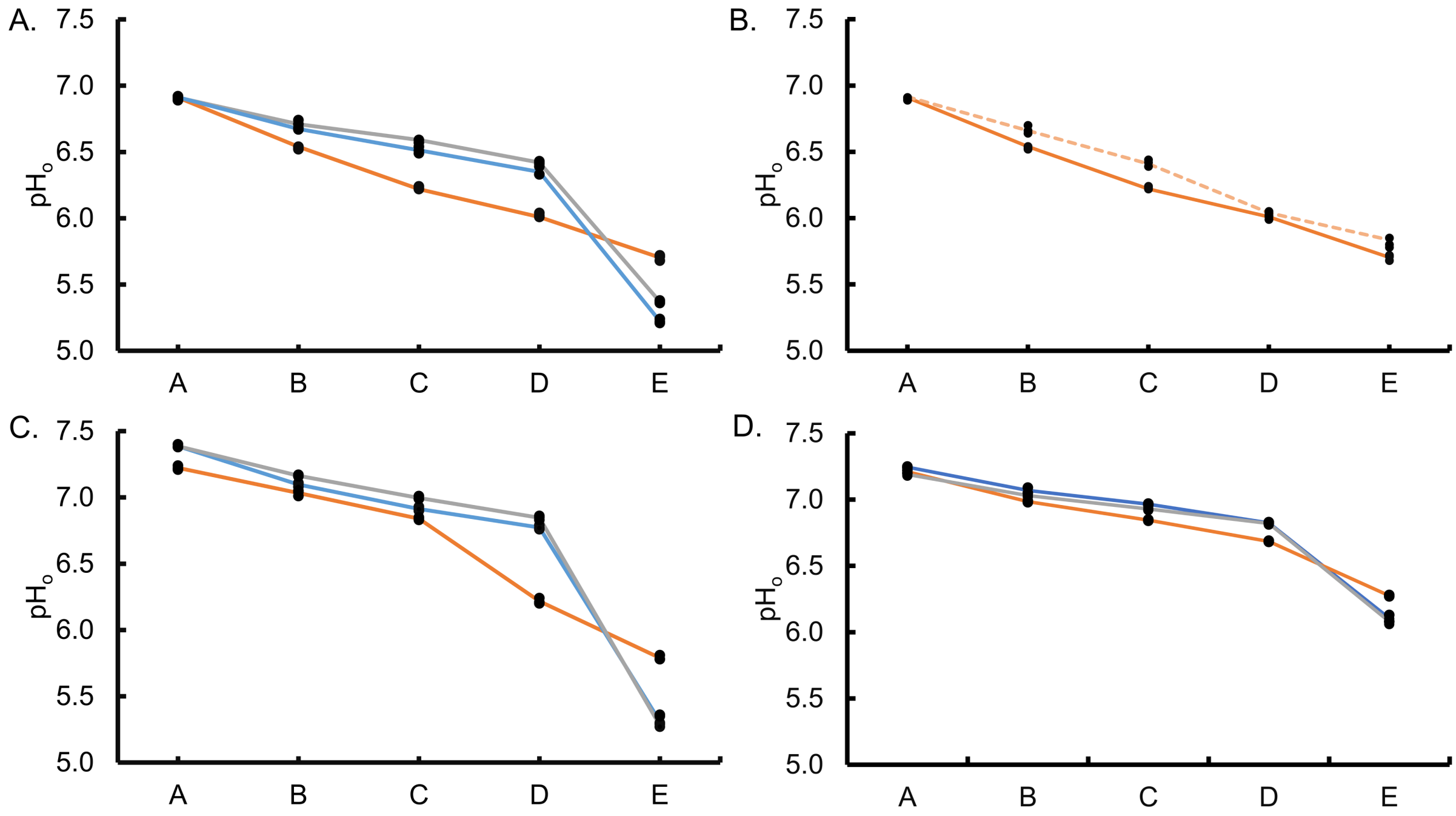

Figure S3. Medium pH during fermentation under different conditions. Shown are the pH_o values determined throughout cultivation of the three strains DH4100 (blue curves), DH4200 (orange curves) and DH4300 (gray curves). Growth conditions included: M9-glucose medium, pH 7 (panel A); M9-glucose medium, pH 8 (panel C); comparison of strain DH4200 grown in M9-glucose medium, pH 7 without (uninterrupted line) and with 10 mM sodium bicarbonate (dotted line) (panel B); and M9-glucose medium, pH 7 supplemented with 50 mM bicarbonate (panel D). The letters A through E within the diagrams, correspond to the growth phases shown in Fig. 1 of the main text. Data points are shown for three biological replicates, with the standard error of the mean shown.

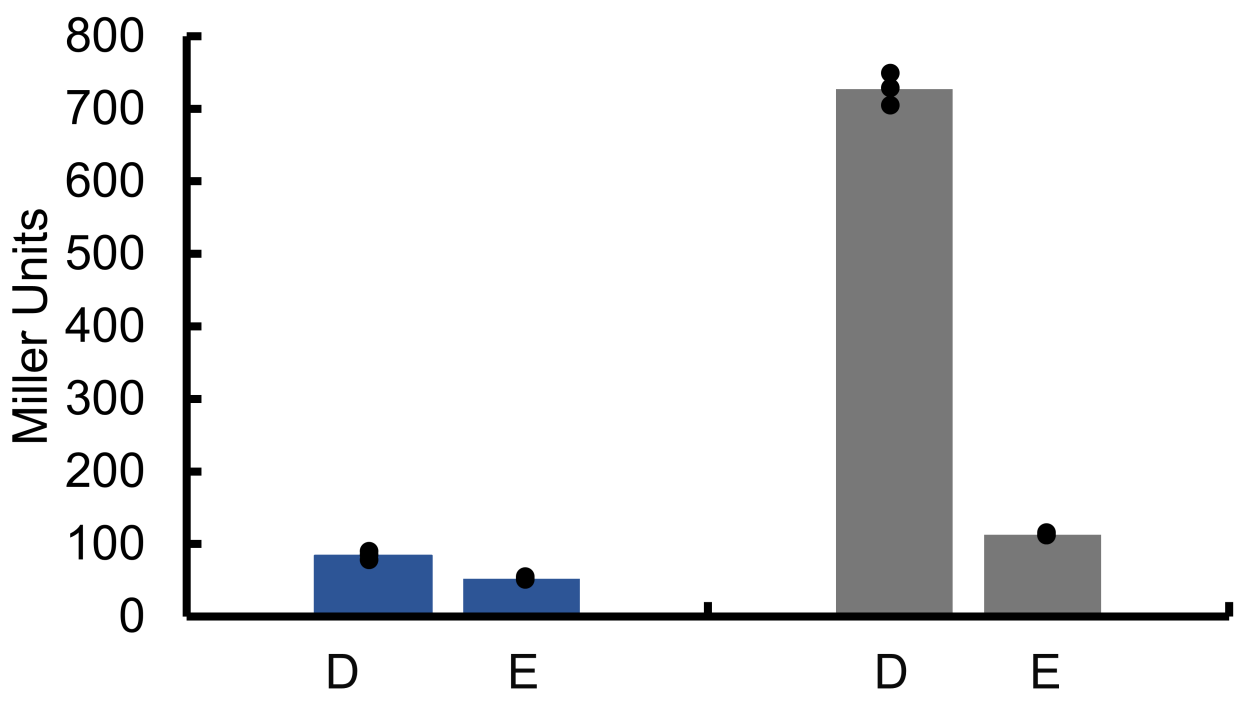

Figure S4. Formate-dependent expression of *$fdhF_P$*::*lacZ* during fermentative growth at pH 8. β-galactosidase enzyme activity of DH4100 (dark blue histograms) and DH4300 (dark gray histograms) after anaerobic cultivation in M9-glucose medium, pH 8. Only phases D and E are shown and are as depicted in Fig. 1 of the main text. Data represent values obtained with three biological replicates for each strain with each data point shown.

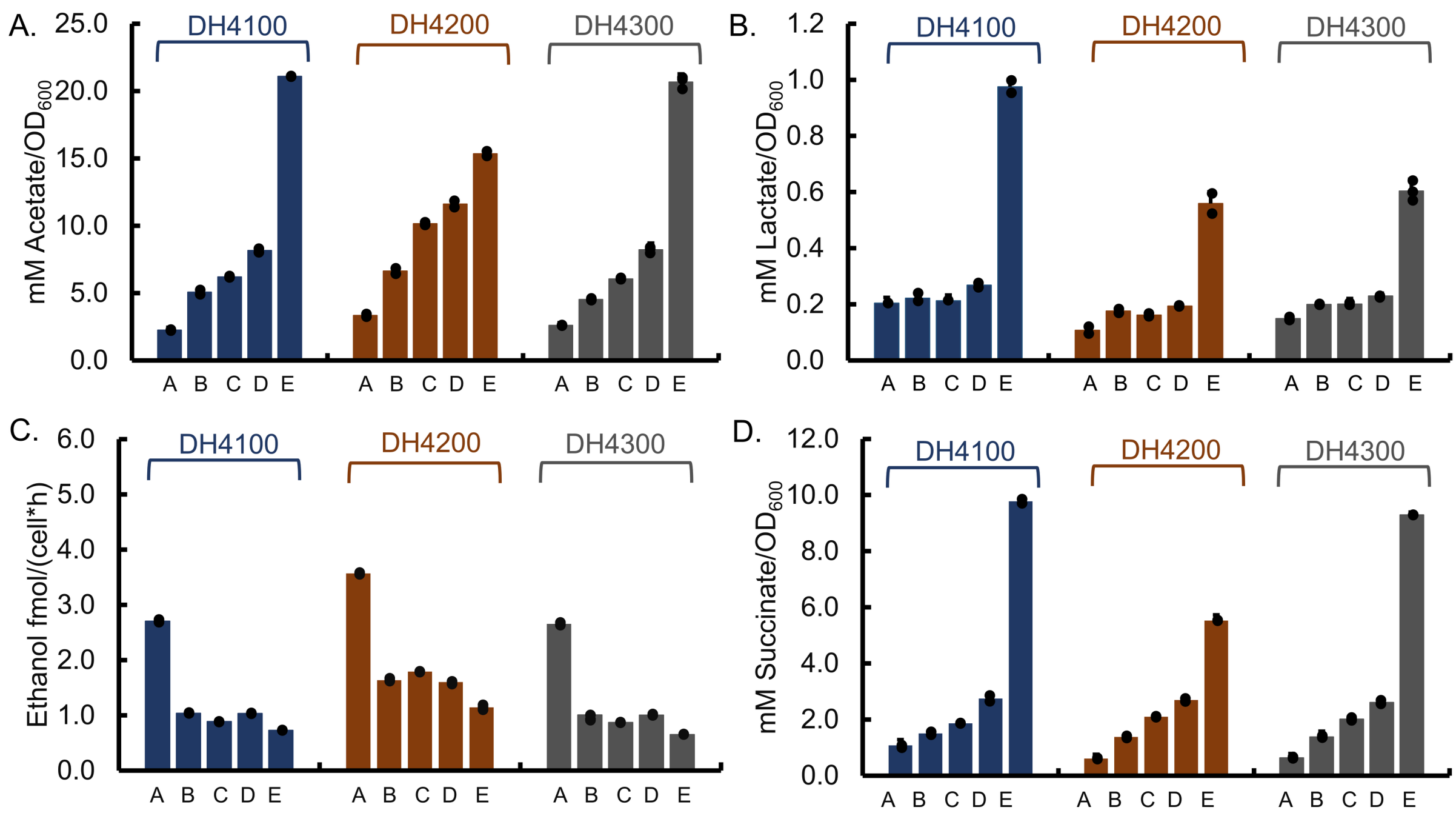

Figure S5. Fermentation product profiles of DH4100, DH4200 and DH4300 during cultivation with 50 mM sodium bicarbonate. The concentrations of acetate (panel A.), lactate (panel B.), ethanol (panel C.) and succinate (panel D.) are shown for each strain during cultivation in M9-glucose minimal medium, pH 7. Parameters were determined at phases A through E and correspond to the growth phases shown in Fig. 1 of the main text. Data points are shown for three biological replicates.

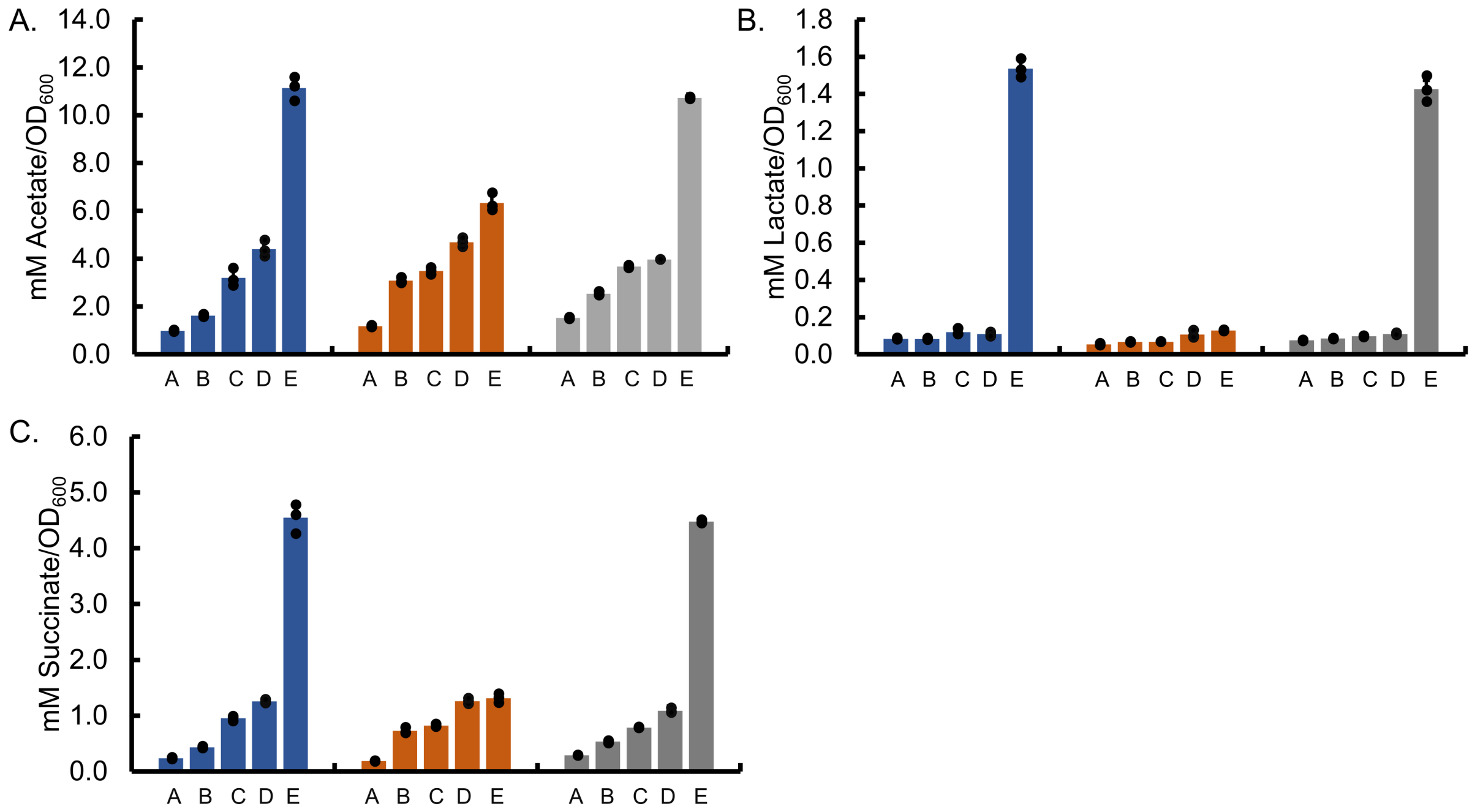

Figure S6. Fermentation product profiles of DH4100, DH4200 and DH4300 during cultivation in M9-glucose medium at pH 8. The concentrations of acetate (panel A.), lactate (panel B.), and succinate (panel C.) are shown for each strain during cultivation in M9-glucose minimal medium, pH 8. Parameters were determined at phases and correspond to the growth phases shown in Fig. 1 of the main text. Data points are shown for three biological replicates.

Summary of Statistical Analyses

Statistics Table S1. Summary of 3-way ANOVA analysis (Jeffrey's Amazing Statistics Program) of the growth curves shown in Fig. 1 of main manuscript. df, degree of freedom.

Source of Variation	Sum of Squares (SS)	df	Mean Square (MS)	F	p	critical F-value
Strain	4,40	2	2,10	1962,00	< 0.001	3,03
pH condition	0,60	2	0,30	251,00	< 0.001	3,03
Phase A - E	45,20	14	3,20	2901,00	< 0.001	1,74
Interaction (Strain x pH Condition)	0,07	4	0,02	1529,00	< 0.001	2,41
Interaction (Strain x Phase A – E)	2,20	28	0,08	69,20	< 0.001	1,53
Interaction (pH Condition x Phase A – E)	1,40	28	0,05	45,90	< 0.001	1,53
Interaction (Strain x pH Condition x Phase A – E)	0,30	56	0,01	4,87	< 0.001	1,41
Error	0,30	270	1,00E-03			
Total	108,94	808				

Statistics Table S2. Summary of 3-way ANOVA analysis (JASP) of the ATP measurements shown in Fig. 2 of main manuscript. df, degree of freedom.

Source of Variation	Sum of Squares (SS)	df	Mean Square (MS)	F	p	critical F-value
Strain	2,78E+06	2	1,39E+06	2,44E+04	< 0.001	3,10
pH condition	2,01E+05	2	1,01E+05	1760,00	< 0.001	3,1
Phase A - E	2,14E+07	4	5,36E+06	9,39E+04	< 0.001	2,47
Interaction (Strain x pH Condition)	1,07E+05	4	2,66E+04	466,00	< 0.001	2,47
Interaction (Strain x Phase A – E)	5,23E+06	8	6,54E+05	1,14E+04	< 0.001	2,03
Interaction (pH Condition x Phase A – E)	6,15E+05	8	7,69E+04	1346,00	< 0.001	2,03
Interaction (Strain x pH Condition x Phase A – E)	4,86E+05	16	3,04E+04	532,00	< 0.001	1,75
Error	5140,00	90	57,11			
Total	6,17E+07	268				

Statistics Table S3. Summary of 2-way ANOVA analysis (JASP) of the pH_i measurements shown in Fig. 3 of main manuscript. df, degree of freedom.

Source of Variation	Sum of Squares (SS)	df	Mean Square (MS)	F	p	critical F-value
Strain	0,56	2	0,28	713,84	< 0.001	3,32
Phase A – E	0,04	4	0,01	28,23	< 0.001	2,69
Interaction (Strain x Phase A – E)	0,19	8	0,02	59,77	< 0.001	2,27
Error	0,01	30	3,93E-04			
Total	0,81	44				

Statistics Table S4. Summary of 2-way ANOVA analysis (JASP) of the β-galactosidase enzyme activity measurements shown in Fig. 4 of main manuscript. df, degree of freedom.

Source of Variation	Sum of Squares (SS)	df	Mean Square (MS)	F	p	critical F-value
Strain	4661943,95	2	2330971,97	4816,74	< 0.001	3,32
Phase A – E	201440,26	4	50360,06	104,06	< 0.001	2,69
Interaction (Strain x Phase A – E)	482310,67	8	60288,83	124,58	< 0.001	2,27
Error	14517,93	30	483,93			
Total	5360212,81	44				

Statistics Table S5. Summary of 3-way ANOVA analysis (JASP) of the external formate determination shown in Fig. 5 of main manuscript. df, degree of freedom.

Source of Variation	Sum of Squares (SS)	df	Mean Square (MS)	F	p	critical F-value
Strain	839,80	2	419,90	3018,00	< 0.001	3,10
pH condition	1613,00	2	806,70	5798,00	< 0.001	3,1
Phase A - E	6098,00	4	1524,00	1,10E+04	< 0.001	2,47
Interaction (Strain x pH Condition)	27,42	4	6,85	49,30	< 0.001	2,47
Interaction (Strain x Phase A – E)	127,80	8	15,98	115,00	< 0.001	2,03
Interaction (pH Condition x Phase A – E)	1297,00	8	162,20	1166,00	< 0.001	2,03
Interaction (Strain x pH Condition x Phase A – E)	107,20	16	6,70	48,20	< 0.001	1,75
Error	12,52	90	0,14			
Total	20245,48	268				

Statistics Table S6. Summary of 2-way ANOVA analysis (JASP) of the fermentation product analysis shown in Fig. 6 of main manuscript. df, degree of freedom.

Source of Variation	Sum of Squares (SS)	df	Mean Square (MS)	F	p	critical F-value
Strain	12,52	2	6,26	111,42	< 0.001	3,32
Phase A – E	125,85	4	31,46	559,84	< 0.001	2,69
Interaction (Strain x Phase A – E)	15,74	8	1,97	35,02	< 0.001	2,27
Error	1,69	30	0,06			
Total	155,80	44				

Source of Variation	Sum of Squares (SS)	df	Mean Square (MS)	F	p	critical F-value
Strain	0,31	2	0,15	102,08	< 0.001	3,32
Phase A – E	6,85	4	1,71	1143,60	< 0.001	2,69
Interaction (Strain x Phase A – E)	2,05	8	0,26	171,35	< 0.001	2,27
Error	0,04	30	1,50E-03			
Total	9,26	44				

Source of Variation	Sum of Squares (SS)	df	Mean Square (MS)	F	p	critical F-value
Strain	9,17	2	4,58	11047,10	< 0.001	3,32
Phase A – E	19,33	4	4,83	11643,89	< 0.001	2,69
Interaction (Strain x Phase A – E)	1,24	8	0,15	372,06	< 0.001	2,27
Error	0,01	30	4,15E-04			
Total	29,74	44				

Source of Variation	Sum of Squares (SS)	df	Mean Square (MS)	F	p	critical F-value
Strain	1,51	2	0,75	33,78	< 0.001	3,32
Phase A – E	83,10	4	20,78	932,56	< 0.001	2,69
Interaction (Strain x Phase A – E)	17,64	8	2,20	98,97	< 0.001	2,27
Error	0,67	30	0,02			
Total	102,92	44				

Statistics Table S7. Summary of 2-way ANOVA analysis (JASP) of the fermentation product analysis shown in Fig. S5 of the Supplementary Material. df, degree of freedom.

Source of Variation	Sum of Squares (SS)	df	Mean Square (MS)	F	p	critical F-value
Strain	8,04	2	4,02	98,14	< 0.001	3,32
Phase A – E	1325,62	4	331,41	8091,53	< 0.001	2,69
Interaction (Strain x Phase A – E)	124,13	8	15,52	378,85	< 0.001	2,27
Error	1,23	30	0,04			
Total	1459,02	44				

Source of Variation	Sum of Squares (SS)	df	Mean Square (MS)	F	p	critical F-value
Strain	0,16	2	0,08	177,60	< 0.001	3,32
Phase A – E	2,04	4	0,51	1153,12	< 0.001	2,69
Interaction (Strain x Phase A – E)	0,22	8	0,03	61,10	< 0.001	2,27
Error	0,01	30	4,42E-04			
Total	2,42	44				

Source of Variation	Sum of Squares (SS)	df	Mean Square (MS)	F	p	critical F-value
Strain	1,99	2	1,00	2073,14	< 0.001	3,32
Phase A – E	12,34	4	3,08	6413,05	< 0.001	2,69
Interaction (Strain x Phase A – E)	0,16	8	0,02	42,37	< 0.001	2,27
Error	0,01	30	4,81E-04			
Total	14,51	44				

Source of Variation	Sum of Squares (SS)	df	Mean Square (MS)	F	p	critical F-value
Strain	10,08	2	5,04	1267,41	< 0.001	3,32
Phase A – E	306,38	4	76,59	19266,27	< 0.001	2,69
Interaction (Strain x Phase A – E)	27,06	8	3,38	850,89	< 0.001	2,27
Error	0,12	30	3,98E-03			
Total	343,64	44				

Statistics Table S8. Summary of 2-way ANOVA analysis (JASP) of the fermentation product analysis shown in Fig. S6 of the Supplementary Material. df, degree of freedom.

Source of Variation	Sum of Squares (SS)	df	Mean Square (MS)	F	p	critical F-value
Strain	4,22	2	2,11	42,87	< 0.001	3,32
Phase A – E	356,47	4	89,12	1810,48	< 0.001	2,69
Interaction (Strain x Phase A – E)	43,22	8	5,40	109,75	< 0.001	2,27
Error	1,48	30	0,05			
Total	405,38	44				

Source of Variation	Sum of Squares (SS)	df	Mean Square (MS)	F	p	critical F-value
Strain	0,83	2	0,42	745,43	< 0.001	3,32
Phase A – E	6,40	4	1,60	2858,57	< 0.001	2,69
Interaction (Strain x Phase A – E)	2,85	8	0,36	636,61	< 0.001	2,27
Error	0,02	30	5,60E-04			
Total	10,10	44				

Source of Variation	Sum of Squares (SS)	df	Mean Square (MS)	F	p	critical F-value
Strain	3,60	2	1,80	300,84	< 0.001	3,32
Phase A – E	58,28	4	14,57	2435,53	< 0.001	2,69
Interaction (Strain x Phase A – E)	17,12	8	2,14	357,77	< 0.001	2,27
Error	0,18	30	0,01			
Total	79,18	44				
